# A Virtual Mixture Approach to the Study of Multistate Equilibrium: Application to Constant pH Simulation in Explicit Water

**DOI:** 10.1371/journal.pcbi.1004480

**Published:** 2015-10-27

**Authors:** Xiongwu Wu, Bernard R. Brooks

**Affiliations:** Laboratory of Computational Biology, National Heart, Lung, and Blood Institute (NHLBI), National Institutes of Health (NIH), Bethesda, Maryland, United States of America; University of Wisconsin-Madison, UNITED STATES

## Abstract

Chemical and thermodynamic equilibrium of multiple states is a fundamental phenomenon in biology systems and has been the focus of many experimental and computational studies. This work presents a simulation method to directly study the equilibrium of multiple states. This method constructs a virtual mixture of multiple states (VMMS) to sample the conformational space of all chemical states simultaneously. The VMMS system consists of multiple subsystems, one for each state. The subsystem contains a solute and a solvent environment. The solute molecules in all subsystems share the same conformation but have their own solvent environments. Transition between states is implicated by the change of their molar fractions. Simulation of a VMMS system allows efficient calculation of relative free energies of all states, which in turn determine their equilibrium molar fractions. For systems with a large number of state transition sites, an implicit site approximation is introduced to minimize the cost of simulation. A direct application of the VMMS method is for constant pH simulation to study protonation equilibrium. Applying the VMMS method to a heptapeptide of 3 ionizable residues, we calculated the pKas of those residues both with all explicit states and with implicit sites and obtained consistent results. For mouse epidermal growth factor of 9 ionizable groups, our VMMS simulations with implicit sites produced pKas of all 9 ionizable groups and the results agree qualitatively with NMR measurement. This example demonstrates the VMMS method can be applied to systems of a large number of ionizable groups and the computational cost scales linearly with the number of ionizable groups. For one of the most challenging systems in constant pH calculation, SNase Δ+PHS/V66K, our VMMS simulation shows that it is the state-dependent water penetration that causes the large deviation in lysine66’s pKa.

## Author Summary

Computer simulation plays an important role to understand molecular systems and has been applied to problems of increasing complexity. Multistate equilibrium is a fundamental concept behind the structure and function of biological systems. Due to the limit in computing resources and lack of good alternative methods, computer simulation has been conducted for systems in a single state, sampling from one state to another to infer equilibrium properties. This sequential approach has been successful in many cases such as protonation equilibrium with implicit solvation model. However, state transition is difficult when explicit solvent is used for more accurate solvation description. Many efforts have been dedicated to overcome this difficulty. Analogous to real multistate systems, we proposed a virtual mixture of multiple states (VMMS) to directly simulate the equilibrium. State transitions are replaced by changes in state molar fractions. Mimicking a test tube environment, all states are simulated in parallel to equilibrate with each other. Application to constant pH simulation in explicit water demonstrates the capability of this method. It is expected that the VMMS method will find more applications in biological problems related to the equilibrium of competing states.

“This is a *PLOS Computational Biology* Methods paper”

## Introduction

Chemical and thermodynamic equilibrium of multiple states is a fundamental phenomenon in biological systems. Typical examples of multiple state equilibria are protonation equilibrium, ligand binding equilibrium, and phosphorylation equilibrium. For example, through the equilibrium of different protonation states, protein can fold or unfold in different pH environments. By changing phosphorylation states, protein can activate or deactivate certain functions. Ligand binding equilibrium controls the inhibition of enzyme activities.

Computer simulation has played an increasingly important role in understanding biological systems. However, due to the limit of computing resources and lack of good alternative methods, equilibrium of multiple states is often studied through simulations of individual states in order to minimize size and complexity of simulation systems. Equilibrium properties are derived indirectly through free energy calculation. For example, ligand binding equilibrium is indirectly studied by calculating binding affinities.

Some methods directly addressing state equilibrium have been developed recently. For example, constant pH simulation methods [1–20] can simulate equilibrium between different protonation states. In these methods, equilibrium between states is simulated through visiting or sampling different states sequentially. In other words, through frequently changing from one state to another, a simulation samples the equilibrium distributions of various states. Such a sequential sampling process works well if the transition between states is easy. For example, with implicit solvation models, transition between protonated and deprotonated states has no difficulty since implicit water reorganizes instantly, and proper sampling can be achieved through, for example, Monte Carlo[5] or replica-exchange[12]. However, when transition between states is difficult, such as in explicit solvent where large barriers exist between states, special efforts are needed to improve the sampling efficiency. Typical examples are the λ-dynamics[14] and the Enveloping Distribution Sampling[19,21–23].

In this work, we present a new simulation approach to address multi-state equilibrium in a parallel way without direct transition between states. All states exist simultaneously in a simulation with populations defined by their molar fractions. The transition between states is implicated by changes of their molar fractions. By avoiding direct transitions between states, this method is designed for problems where transitions between states are difficult, such as deprotonation in explicit solvent. We call this new approach the virtual mixture of multi-states (VMMS) method, because this method simulates multiple states explicitly and treats explicit states as though in a virtual mixture to equilibrate with each other.

## Results

The VMMS simulation method uses multiple techniques to achieve efficient simulation. Through several examples, we validate the techniques and demonstrate the application of the VMMS method in the study of protonation equilibrium.

### Free energy calculation with IPS

Using the IPS potential for long range energy calculation is a key factor for VMMS efficiency. The IPS potential converts long-range interactions to pairwise interactions within a certain range, typically within a cutoff. This cutoff region is called the local region. Everywhere beyond the local region is called the remote region. Interactions with the remote region are replaced by interactions with the isotropic periodic image of the local region. The summation over the IPS image is an analytic function of the distance between particles within a local region. Ewald sum uses lattice images to calculate long range interactions. It has contributions from all atom pairs and depends not only on the distances, but also on the orientations of all atom pairs. Due to these complexities, Ewald summation needs special numerical methods such as the Particle Meshed Ewald (PME) technique to improve efficiency. Apparently, IPS and PME are based on similar concepts, but with different local regions and images. It has been demonstrated that IPS produces very similar results as PME [24–29]. However, PME is not pairwise and to separate solute and solvent interactions requires multiple PME calculations, thus, making it less suitable for the VMMS simulation.

**Fig 1:**
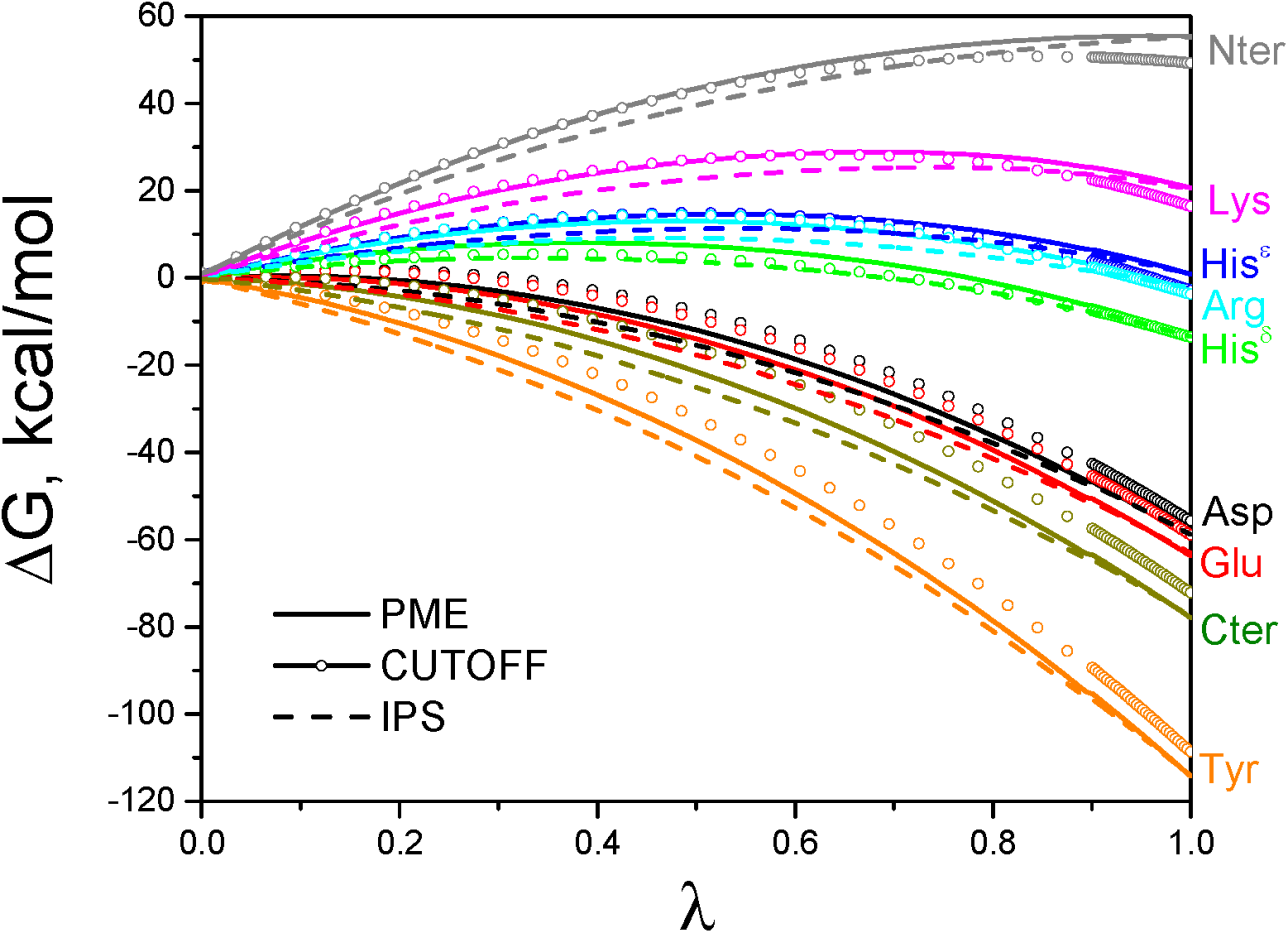
Deprotonation free energy profiles during the thermodynamic integration calculations. The PME and IPS results are shown as solid and dashed lines, respectively. The cutoff results are shown with open circles. Results for different residues are colored differently as labeled.

Although many cutoff methods are pairwise, they cannot provide accurate free energies, which is crucial for the study of state equilibrium. IPS is as simple as any cutoff method, but, as accurate as PME [25–32]. To demonstrate the accuracy of IPS potential, we calculated the deprotonation free energies of the model compounds. The free energy profiles during the calculations are shown in Fig 1. For comparison, Fig 1 also shows the results from PME and another cutoff method.

The deprotonation free energies were calculated through thermodynamic integration with the PERT module of CHARMM. Each of the model compounds was dissolved in a box of TIP3P water. The box size was 31.1×31.1×31.1 Å^3^. The cutoff method[33] used force shift for electrostatic potential and energy switch for Lennard-Jones potential with r_on_ = 10 Å and r_off_ = 12 Å. The 3D IPS used a local radius of *R*_*c*_ = 12Å. For PME, a real space cutoff of 12 Å was used and the grid dimension for Fast Fourier transform (FFT) calculation was 32×32×32. There were total 190 λ windows, Δλ = 0.01 for 0≤λ≤0.9 and Δλ = 0.001 for 0.9<λ≤1. For each window a 10 ps LD simulation was performed with a friction constant of 1/ps.

From Fig 1 we can see that the IPS results converge well with the PME results at λ = 1, with an average deviation of 0.37 kcal/mol. The cutoff results have an average deviation of 4.4 kcal/mol from the PME results. These results demonstrate that IPS is an efficient and competitive alternative to PME when pairwise properties are needed. The cutoff methods can provide pairwise terms, but their deviation from PME makes them a poor choice for quantitative studies.

Note that at intermediate λ the IPS and the PME results are differ, but at the significant end points (λ = 0 and λ = 1) the variation of free energy differences between IPS and PME is very small. During the free energy calculation, the energy at intermediate λ was a combination of the two end states. The difference at intermediate λ values is due to the different treatments of boundary interactions in the two methods. What matters is the free energy difference between the end states, which is used to determine the state equilibrium.

### VMMS ensemble distribution

Reweighting a VMMS conformation distribution to obtain the pure state conformational distributions is the key to simulate multistate equilibrium. The partition function of the VMMS ensemble, Eq (6), describes the VMMS conformational distribution and provides a reweighting relation, Eqs (9) and (10), to convert a VMMS distribution to a pure state distribution. Here we use two simple systems to demonstrate this reweighting relation.

**Fig 2:**
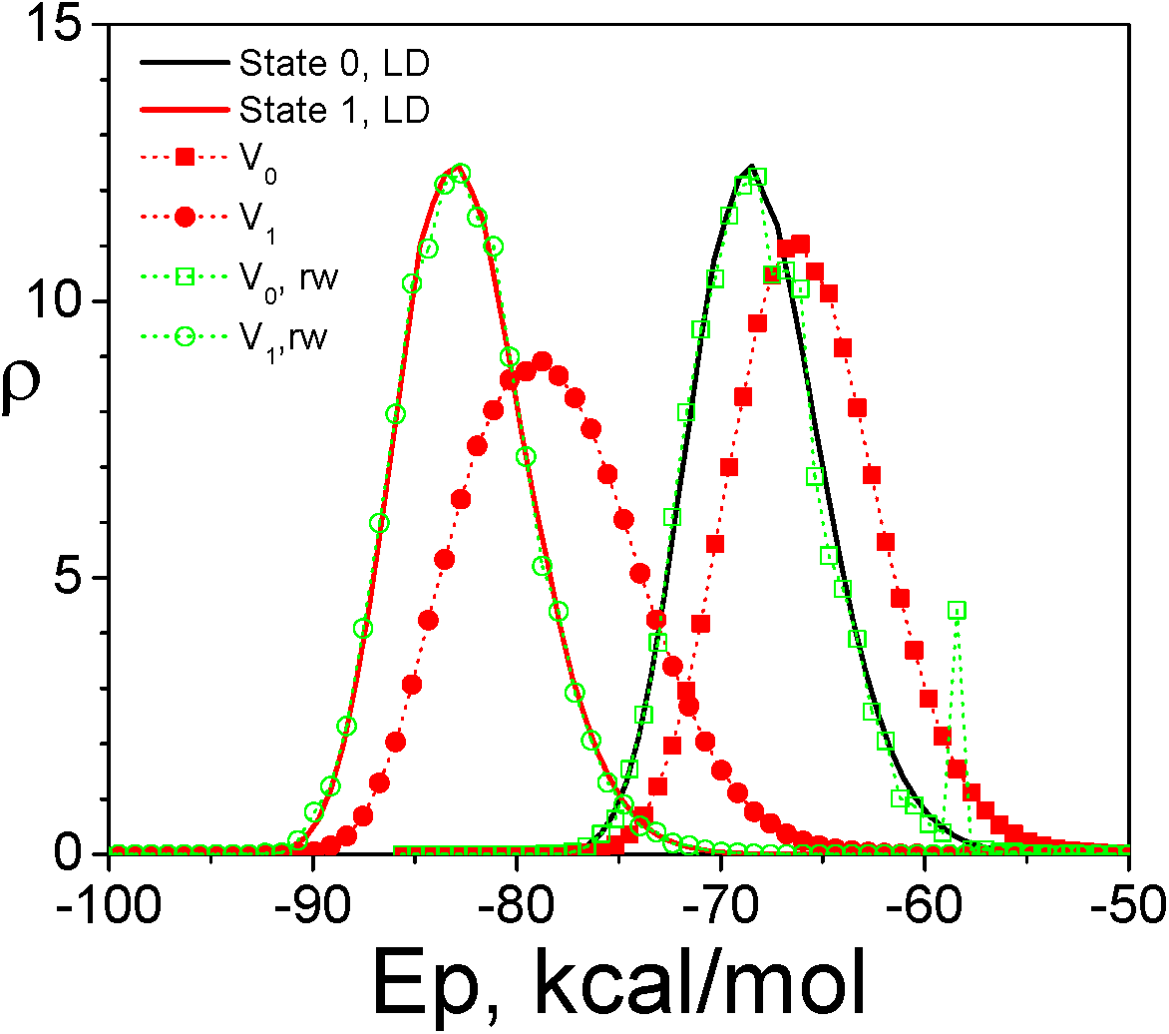
Energy distributions of the ASP model compound with the GB implicit solvation model in different states. The solid lines are the protonated state (state 0, black) and deprotonated state (state 1, red). The red dotted lines with filled symbols are in the V_0_ (square) state and V_1_ (circle) state. The green dotted lines with open symbols are the reweighted results in the V_0_ (square) and V_1_ (circle) states.

First, we use a model compound, ACE-ASP-NME, with the generalized Born (GB) implicit solvation model[34] to examine the VMMS ensemble. With an implicit solvation model, the VMMS system contains only the solute in two charge states, the protonated state (state 0) and the deprotonated state (state 1). Fig 2 shows the energy distributions from the VMMS simulation at *x*_H_ = *x*_D_ = 0.5 and from the Langevin dynamics (LD) simulations of the pure states. As can be seen, the two ensembles have quite different energy distributions, with the VMMS ensemble shifting toward higher energy. After reweighting with Eq (10), the VMMS results match very well with the LD results for the pure states, validating the reweighting relation, Eq (10) and verifying indirectly that Eq (6) describes the conformational distribution of the VMMS ensemble. It should be noted that the reweighting process often has large noise at high energy region. A peak around -58 kcal/mol in the reweighted distribution (green line) is due to such noise.

Next, we examine a very simple model system with explicit solvent. Because a regular system with explicit solvent is often overwhelmed by solvent-solvent interactions, making it difficult to see the difference between a VMMS state and a pure state, we built a model solution with just one water molecule as the solute and 10 water molecules as the solvent in a cubic periodic box for each state. To make the two states more distinct, the solute charges were changed to have *q*_O_ = 2.64*e* and *q*_H1_ = 0 and *q*_H2_ = -0.64 *e* for state 0 and *q*_O_ = -2.64 *e*, *q*_H1_ = 0.64 *e*, and *q*_H2_ = 0 for state 1. As a result, the solute had a net charge of +2*e* in state 0 and a net charge of -2*e* in state 1. The solvent charges were scaled by 0.1 to have *q*_O_ = -0.0834 *e* and *q*_H1_ = *q*_H2_ = 0.0417 *e*. The solvent charge scaling made this solution a homogeneous system at T = 300 K and made the two states distinct in energy distribution. The cubic box size was 15.55×15.55×15.55 Å^3^. The 3D IPS potentials, Eqs (11)–(16), with a cutoff radius of 7.5 Å, which was less than half of the box size, were used for both Lennard-Jones and electrostatic interactions.

**Fig 3:**
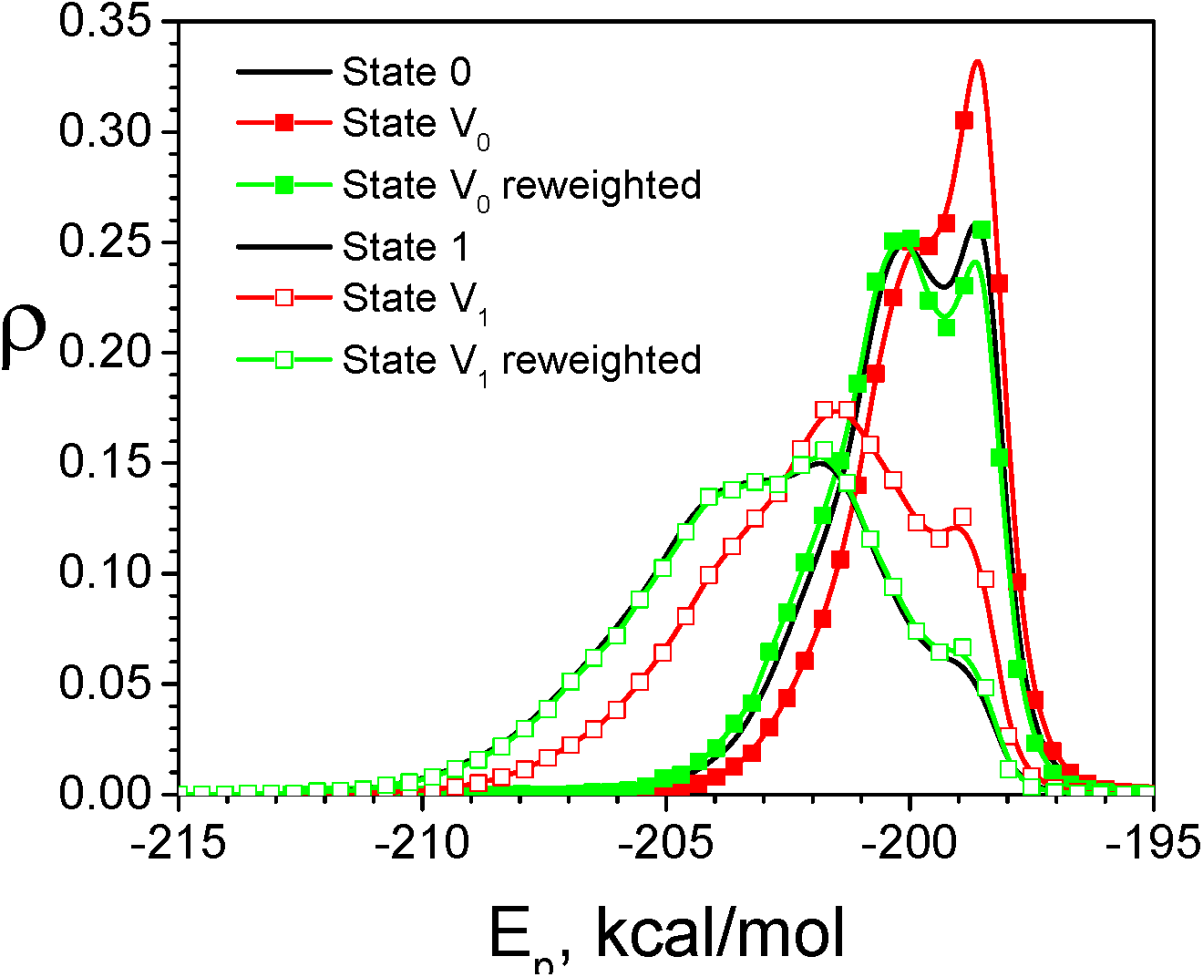
Energy distributions of the model HOH solution in the canonical ensemble and in the VMMS ensemble. The black lines are the LD simulation results for state 0 (solid) and state 1 (dashed). The red lines with squares are the VMMS simulation results for state V_0_ (filled) and state V_1_ (open). The green lines with circles are the reweighted VMMS simulation results for state V_0_ (filled) and state V_1_(open).

We performed a VMMS simulation at *x*_H_ = *x*_D_ = 0.5 and LD simulations for both pure states. All simulation were done at T = 300K and ξ = 1/ps. The energy distributions of this model solution are shown in Fig 3. The VMMS distributions are quite different from the LD distributions for both states. After reweighting, the VMMS energy distributions match very well with the LD distributions. This example validates the VMMS reweighting function, Eq (10), for explicit solvent.

### VMMS simulations of model compounds

**Fig 4:**
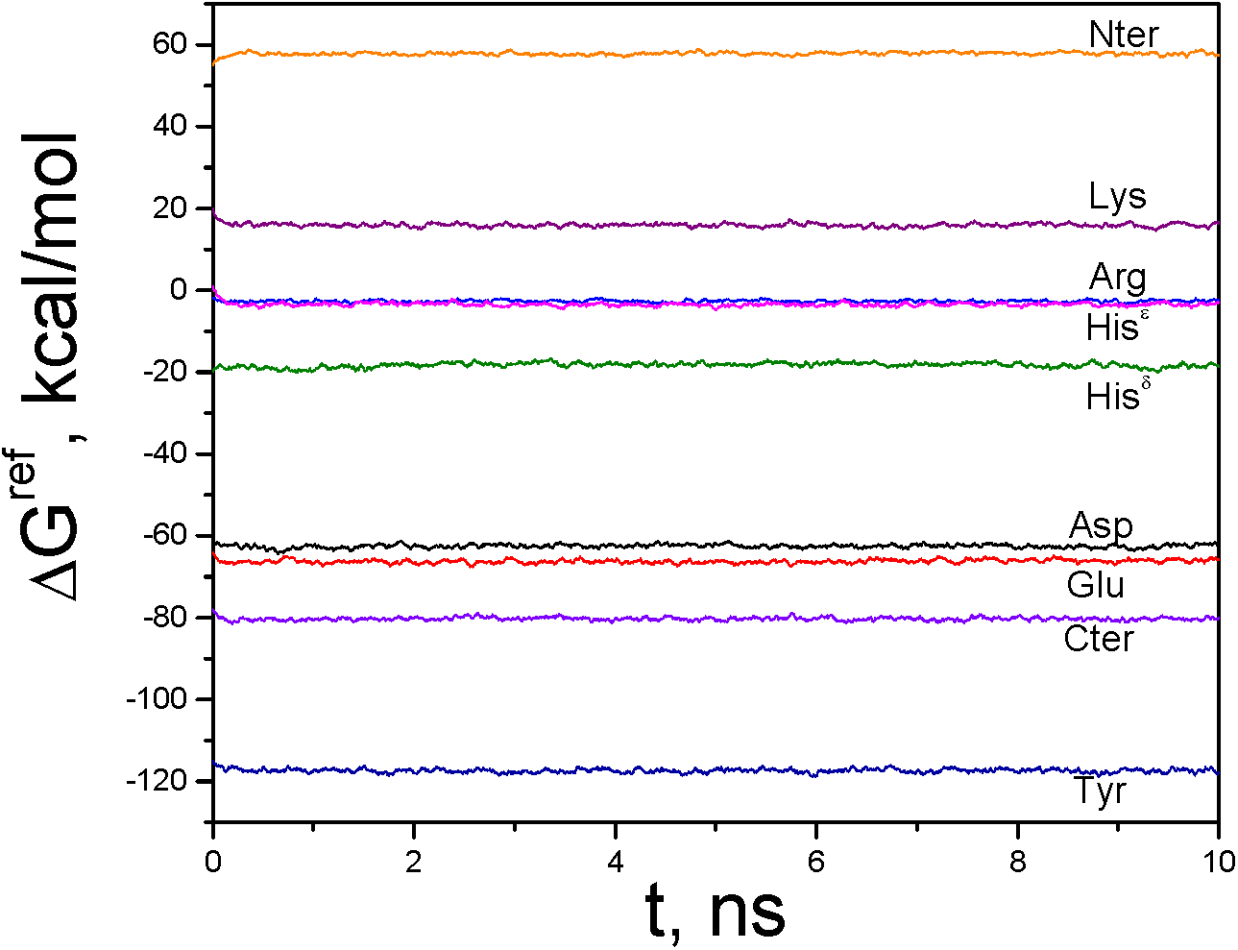
Deprotonation free energies evaluated during the equal-molar VMMS simulations of the model compounds.

The VMMS simulation converts a state transition problem to a free energy calculation between the two states and pushes all contributions, such as chemical bonding and proton creation, into a reference value derived from the experimental pKa and simulated free energy difference of model compounds, as described by Eq (24). Therefore, we need to estimate the free energy difference of the modeling compounds before we can study state equilibrium of interested systems. To calculate the deprotonation free energies of the model compounds, we performed equal-molar VMMS simulations at fixed composition, *x*_H_ = *x*_D_ = 0.5. Fig 4 shows deprotonation free energies evaluated during the equal-molar VMMS simulations. The deprotonation free energies and the experimental pKas of these model compounds are used as reference free energies and reference pKas of the corresponding titration sites and are listed in Table 1.

**Table 1:** Deprotonation free energies calculated using equal-molar VMMS simulations and pKa values calculated from constant pH VMMS simulations. Experimental pKa values are listed as reference pKas.

Compounds	ΔG^ref^, kcal/mol	pKa(VMMS)	pKa^ref^(exp)[12]
ASP	-62.4±0.4	4.0±0.2	4.0
GLU	-66.2±0.4	4.4±0.2	4.4
HIS^δ^	-18.0±0.4	6.4±0.4	6.5
HIS^ε^	-3.4±0.4	7.4±0.4	7.1
ARG	-2.6±0.3	12.4±0.3	12.5
LYS	15.9±0.4	10.4±0.2	10.4
TYR	-117.4±0.5	9.6±0.2	9.6
Nter	57.9±0.3	7.4±0.5	7.5
Cter	-80.2±0.4	3.8±0.6	3.8

**Fig 5:**
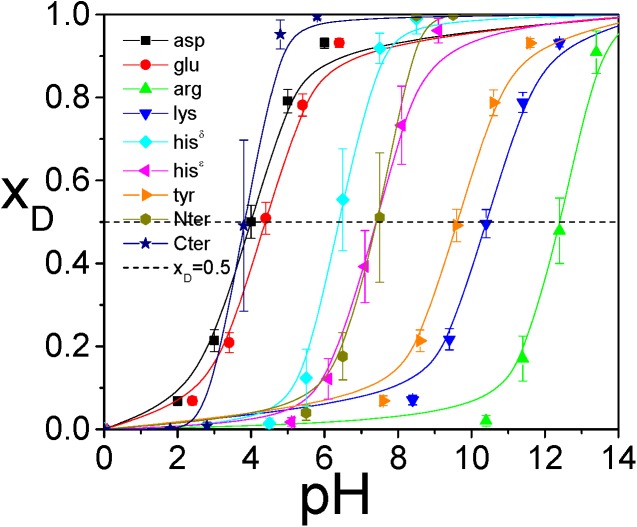
The titration curves of the model compounds obtained from the VMMS simulations.

Using these reference deprotonation free energies, we performed VMMS simulations for the model compounds at constant pH values to identify the equilibrium molar fractions. Fig 5 shows the molar fractions of the deprotonated states as functions of pH. The pKa should be the pH where *x*_D_ = 0.5. The pKa values estimated this way are listed in Table 1. As can be seen, the pKa estimated from the VMMS simulations agree well with the experimental values. Large fluctuations in the pKa results are caused by the large fluctuation in molar fractions and the short simulation lengths.

### VMMS simulations of a heptapeptide with 3 titration sites

Now we are ready to apply the VMMS method in constant pH simulations. Here, we choose a peptide of 7 residues with a sequence: ACE-SDNKTYG-NME. This heptapeptide was derived from OMTKY3 and has been studied experimentally[35] and computationally[3,15,35]. The peptide was dissolved with 966 TIP3P water molecules in a 31.1Å× 31.1Å× 31.1Å cubic box.

First, we present the results with all titration sites explicitly simulated. There are three ionizable residues, D, K, and Y and a total of 2^3^ = 8 titration states. For easy discussion, we denote the 8 states by the states of these three residues, H for a protonated residue and D for a deprotonated residue. For example, HHH stands for a state where all the three residues are in protonated states and DHD stands for a state where residues D and Y are in the deprotonated state and residue K in the protonated state.

As described in the method section, with 3 explicit sites, the VMMS system contains 8 simulation boxes with each state in one box. The peptide in the 8 boxes has the same conformation and moves the same way, but water molecules in each box interact with the peptide in one of the eight states and are independent of water in other boxes.

**Fig 6:**
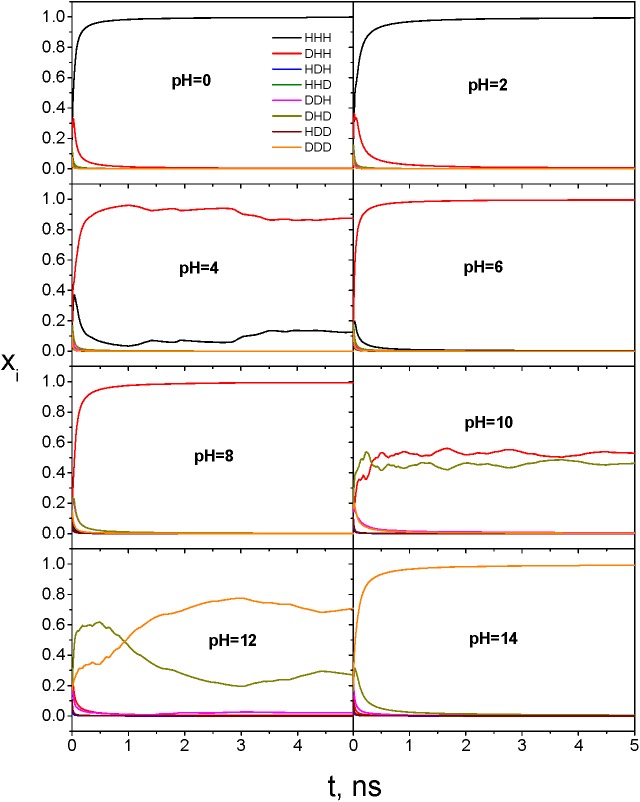
State molar fractions of the heptapeptide, Ace-SDNKTYG-NME, during constant pH VMMS simulations. pH values are labeled in each panel. All states have an initial molar fraction of 0.125.

The VMMS simulations were performed for the peptide at constant pH of 0, 2, 4, 6, 8, 10, 12, and 14. All states initially had a molar fraction of 0.125 and were allowed to change to reach equilibrium. The state molar fractions during these simulations are shown in Fig 6. At pH = 0 and 2, all states approached zero except state HHH. At pH = 4, state DHH became dominant followed by state HHH. At pH = 6 and 8, DHH was the only noticeable state. At pH = 10, DHH and DHD were the competitive dominant forms. At pH = 12, DDD became the dominant form with significant amount of DHD. At pH = 14, DDD was the only dominant form.

**Fig 7:**
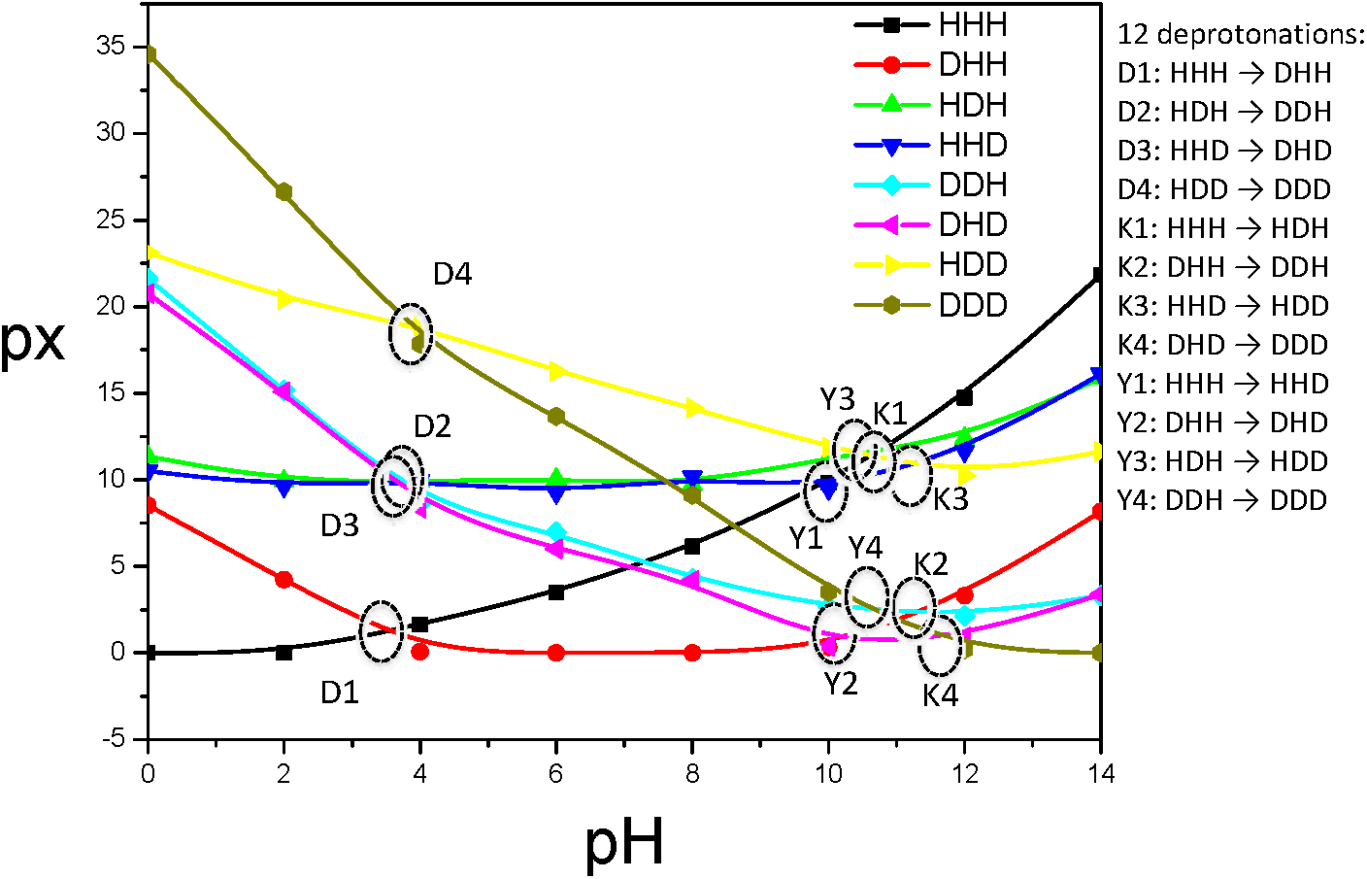
State molar fractions obtained from the constant pH VMMS simulations. There are 12 deprotonations and their pKa can be found from the intersection pH between curves of the protonated and deprotonated states, where *x*_H_ = *x*_D_. The overall pKa will be determined by the dominant forms, i.e., the lowest intersections.

The equilibrium molar fractions of all states are plotted against pH in Fig 7. There are in total 12 deprotonation processes between these 8 states. The pKa of these deprotonation processes can be determined by the pH values where the molar fractions of both the protonated state and deprotonated state are equal. There are four deprotonation processes for each residue and their equal molar positions are circled in Fig 7. As can be seen, the intersections of protonated state and deprotonated state are around pH = 4 for Asp, around 11 for Lys, and around 10 for Tyr. The pKa values of the ionizable residues are determined by the most dominant states and the results are listed in Table 2. These results are reasonably close to the pH-REMD and CpH results obtained with an implicit solvation model[15].

**Table 2:** pKa of the ionizable residues in the heptapeptide from the VMMS simulations and other studies.

residues	VMMS	VMMS–1	pH-REMD[15]	CpHMD[15]	Exp[35]
asp	3.6±0.5	4.1±0.3	3.6±0.2	3.7±0.2	3.6
lys	11.5±0.5	11.1±0.4	10.6±0.1	10.6±0.1	
tyr	10.2±0.5	9.9±0.5	10.1±0.1	9.9±0.1	

Next, we show the results of VMMS–1 simulations where at any moment only one site was explicit and the other two sites were implicit. As described in the method section, when a site is implicit, the atomic charges of this site will be calculated with Eq (27) based on the atomic charges in protonated and deprotonated states and their molar fractions. When a site changes from implicit to explicit, atomic charges return to the values in the corresponding states.

In the VMMS–1 simulations, each site was explicitly simulated for 10 ps and then became implicit. The first 2 ps simulation was used for equilibrium and the following 8 ps were used to evaluate free energy differences and to update molar fractions of explicit states. After becoming implicit, the ionizable sites maintain their state molar fractions until they become explicit again.

**Fig 8:**
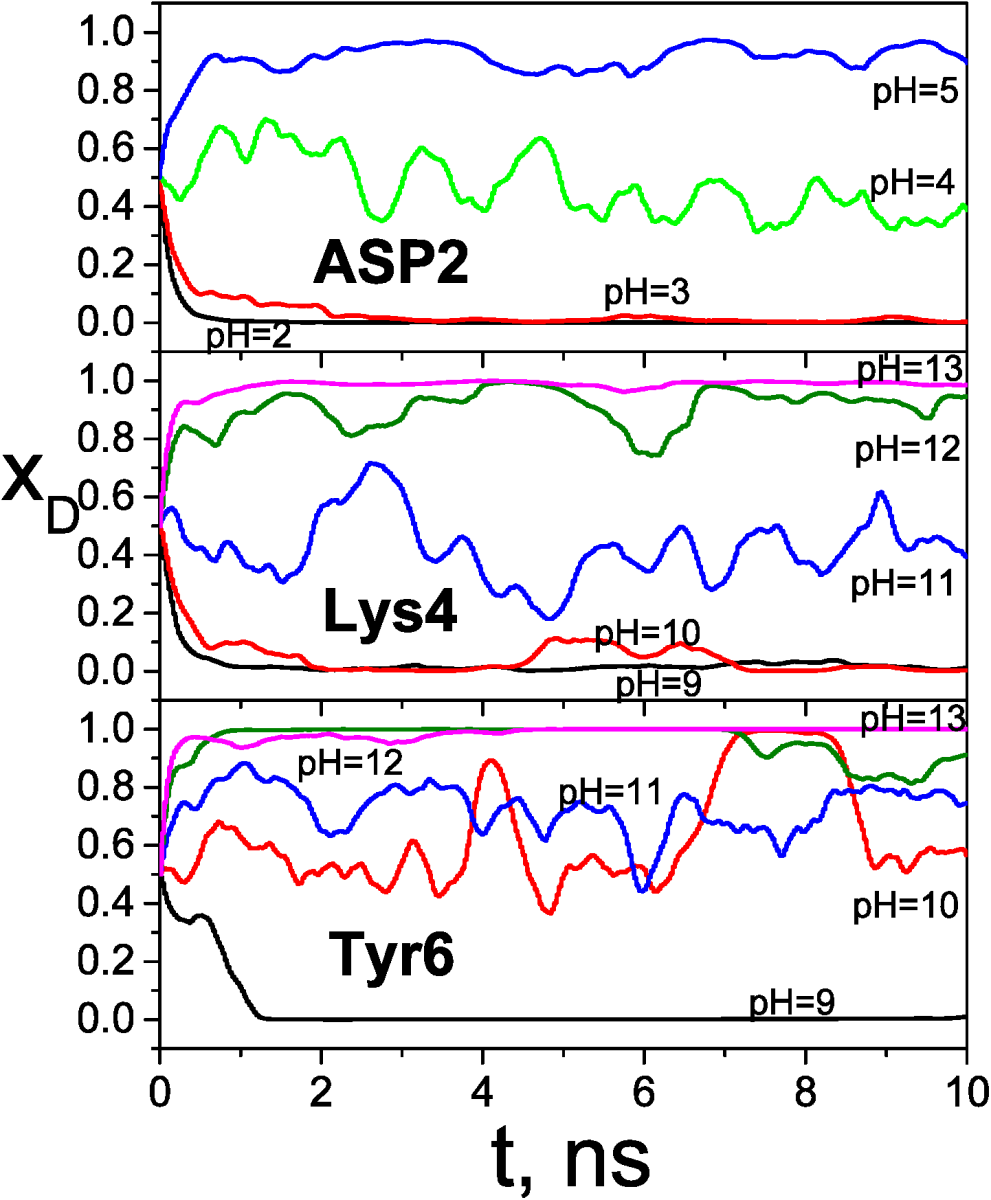
The molar fractions of deprotonated states during the VMMS–1 simulations of the heptapeptide.

Fig 8 shows the molar fractions of the three ionizable sites during the VMMS–1 simulations at various pH values. All simulations started with the deprotonated molar fractions, *x*_D_, of 0.5 for all three sites. The molar fractions of these three sites responded differently to pH values. Large fluctuations in molar fractions were observed when the pH value was close to the pKa value.

**Fig 9:**
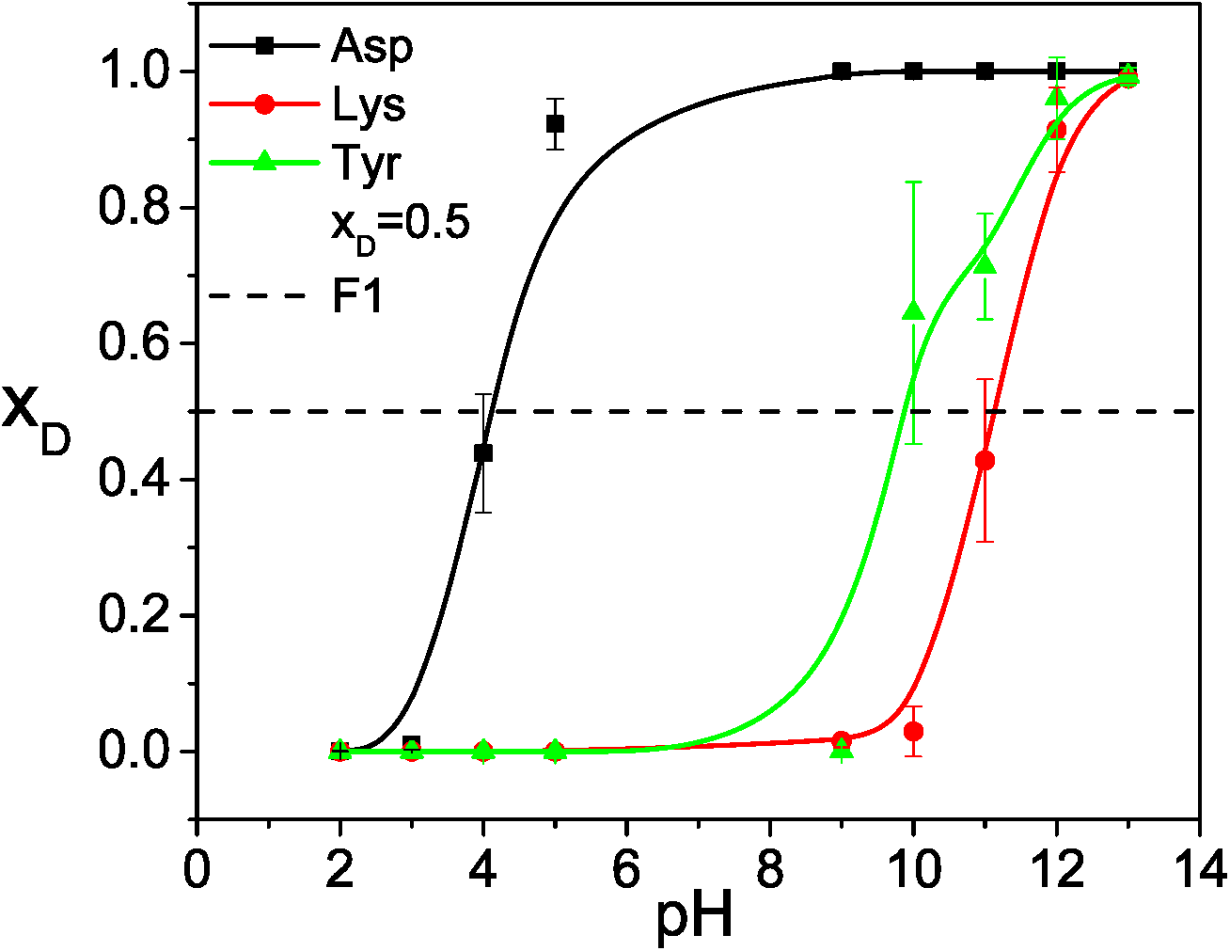
The molar fractions of deprotonated states as functions of pH for the heptapeptide obtained from the VMMS–1 simulations.

Fig 9 plots the molar fractions as functions of pH for the three ionizable residues. The pKa values determined from the pH values where *x*_D_ = 0.5, are listed in Table 2. Within the statistic ranges, the VMMS and VMMS–1 results agree with each other. The consistency of the results from the fully explicit VMMS and the partially implicit VMMS–1 demonstrates that the implicit site approximation is acceptable for this system.

### Mouse epidermal growth factor with 9 ionizable groups

To further illustrate the application of the VMMS method in constant pH study, we applied the VMMS method to mouse epidermal growth factor (EPG) in explicit water, for which pKa’s of 9 ionizable groups have been determined by NMR[36] (Table 3). It is impractical to explicitly simulate all protonation states of these 9 ionizable groups. Instead, we performed VMMS–1 simulations where only one site at a time was explicit and the rest were implicit.

**Table 3:** pKa of the ionizable residues in mouse epidermal growth factor from the VMMS simulations and other studies.

residues	group	VMMS–1	NMR[36]
Asn1	α-NH_3_	9.5±0.5	7.7±0.1
Asp11	β-COOH	2.7±0.4	3.9±0.05
His22	imidazole	7.5±0.5	6.8±0.1
Glu24	γ-COOH	4.6±0.3	4.1±0.1
Asp27	β-COOH	4.5±0.5	4.0±0.1
Asp40	β-COOH	3.5±0.5	3.6±0.1
Asp46	β-COOH	5.5±0.5	3.8±0.1
Glu51	γ-COOH	5.4±0.5	~4
Arg53	α-COOH	4.6±0.4	3.5±0.1

EPG has 53 residues. Its structure is available in PDB (PDB code: 1EPI). This structure was dissolved in a 62.21×46.65×46.65 Å^3^ water box with 4273 TIP3P water molecules. Simulations were performed at constant temperature of 300 K and constant volume. 3D IPS with a local region radius of 12 Å was used for both Lennard-Jones and electrostatic interactions. Every ionizable group was explicitly simulated for 10 ps in the sequential order.

**Fig 10:**
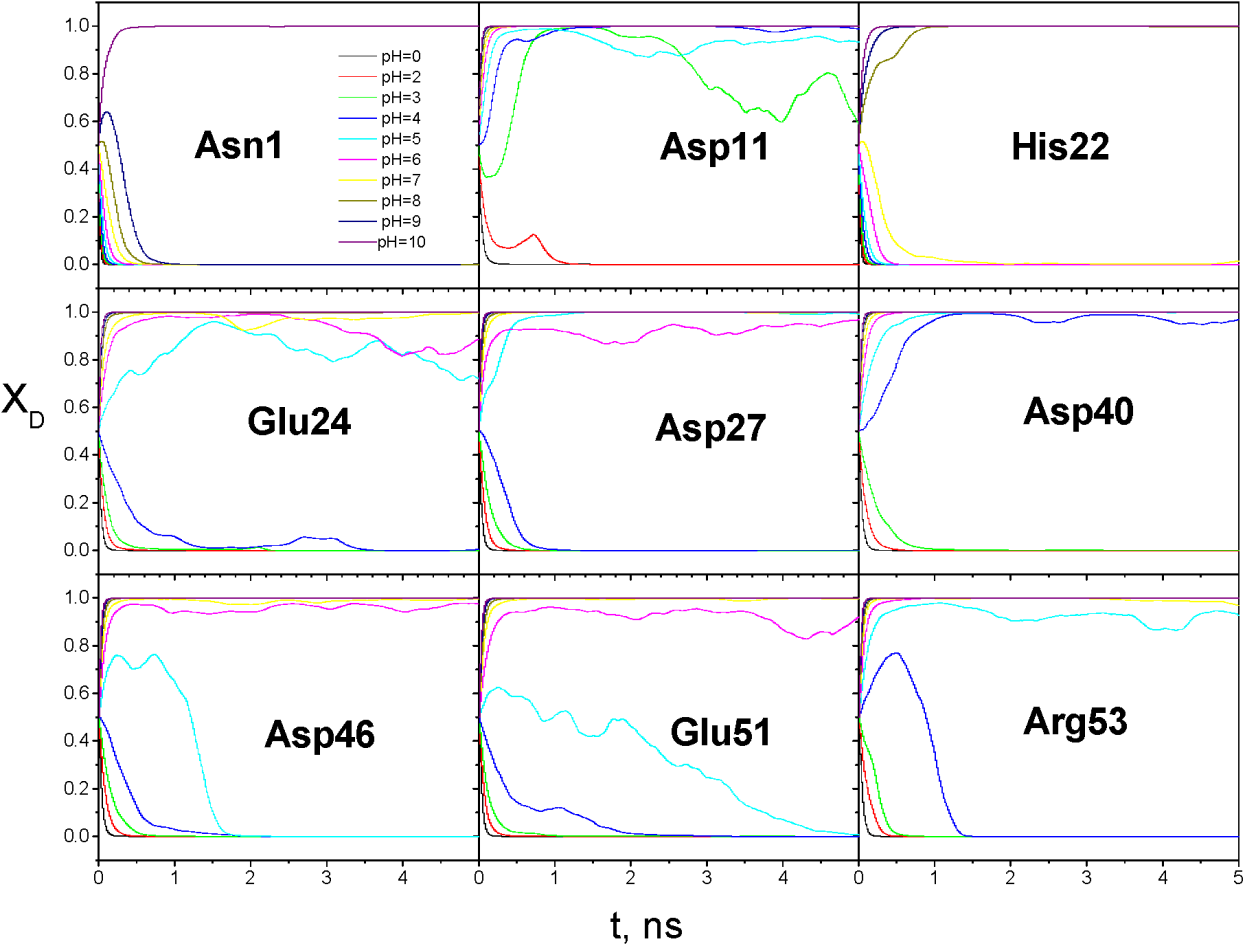
The molar fractions of deprotonated states for the 9 ionizable groups of EPG during the VMMS–1 simulations.

The VMMS–1 simulations were performed at 10 pH values: 0, 2, 3, 4, 5, 6, 7, 8, 9, and 10. The molar fractions of the 9 ionizable groups are shown in Fig 10. As can be seen, starting from 0.5, the deprotonated molar fractions of these ionizable groups changed gradually to approach their equilibrium values. While 5 ns was not long enough for all states to reached their equilibrium molar fractions, the results show that the molar fractions of different group responded differently to the pH changes and provide rough estimates of the pKas.

For the N-terminal group at Asn1 the deprotonated molar fraction approached 0 when pH<9 and 1 when pH = 10. The four Asp residues, Asp11, Asp27, Asp40, and Asp46, were all different from each other. For Asp11, the titration point falls between pH = 2 and pH = 3. For Asp27, the titration point is between pH = 4 and pH = 5. Whereas for Asp40, it is between pH = 3 and pH = 4, and for Asp46, it is between pH = 5 and pH = 6.

**Fig 11:**
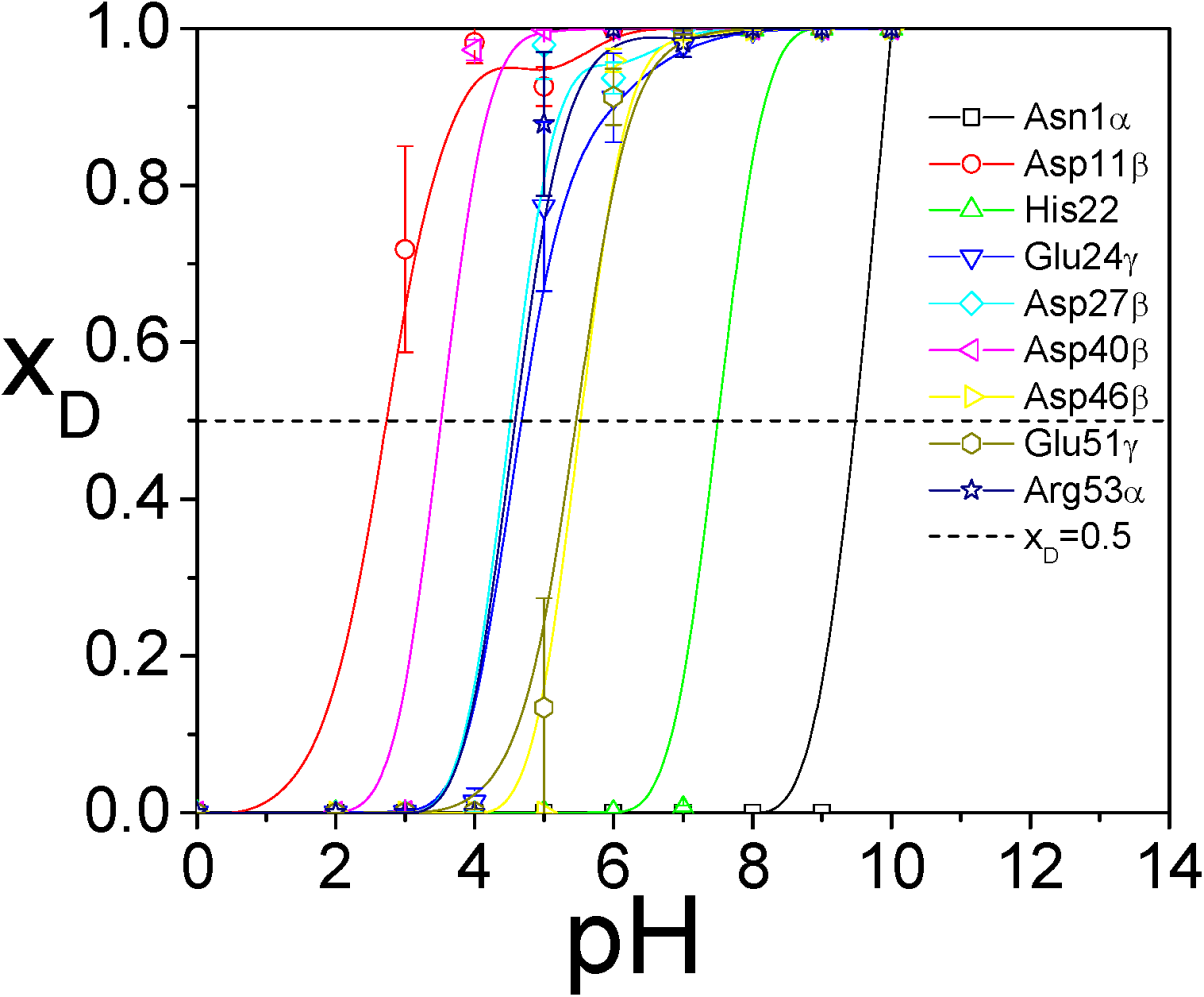
The titration curves of the 9 ionizable groups of EPG as functions of pH values obtained from the VMMS–1 simulations.

We used the average molar fractions between 2 and 5 ns to plot the titration curves for each ionizable group in Fig 11. From the curves we can read the pKa values of these 9 groups, which are listed in Table 3. The agreement with NMR results is qualitatively good. To increase the accuracy, we need much longer simulations. Also, the simulation setup may also affect the results, such as salt concentration, force field, water model, etc. From this example, we demonstrated that the VMMS–1 method can be applied to systems of many ionizable sites and the computing cost scales linearly with the number of ionizable sites.

### VMMS simulation of SNase Δ+PHS/V66K in explicit water

To illustrate the capability of the VMMS method, we chose to simulate one of the most challenging systems in constant pH simulation, SNase Δ+PHS/V66K (PDB: 3HZX). This system is challenging because the Lys66 is buried inside the protein and has a large deviation in pKa from typical lysine residues. This system has been the focus of many studies[37–39]. Experimentally the pKa of this lysine is 5.6, very different from typical pKa of 10.4 for lysine.

In our VMMS simulation, only K66 has explicit protonated and deprotonated states. This protein was dissolved with 2830 TIP3P water in a 46.65×46.65×46.65 Å^3^ cubic box. 100 ns VMMS simulation was performed at constant molar fraction of *x*_H_ = *x*_D_ = 0.5.

**Fig 12:**
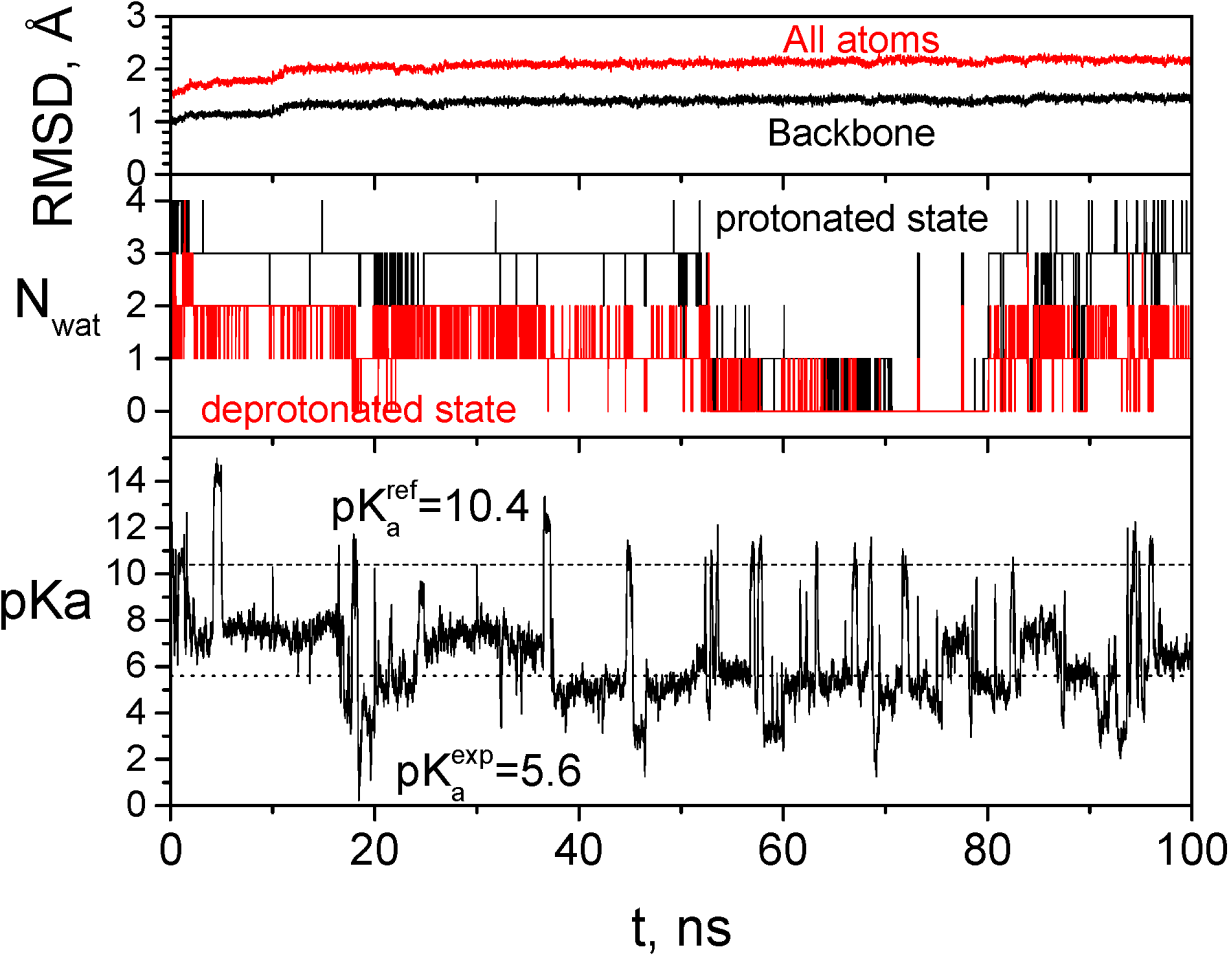
The pKa of K66 and conformational properties of SNase Δ+PHS/V66K during the VMMS simulation.

The pKa of K66 obtained from the simulation is shown in Fig 12. Clearly, the pKa had large fluctuations, representing environment changes around K66. The pKa quickly reached 7 in 3 ns and remained there for more than 10 ns. The pKa dropped to below 4 at around 20 ns, then came back to above 7 at about 30 ns. Before 40 ns the pKa dropped to below 5.6 and fluctuated around 5.6 afterward.

**Fig 13:**
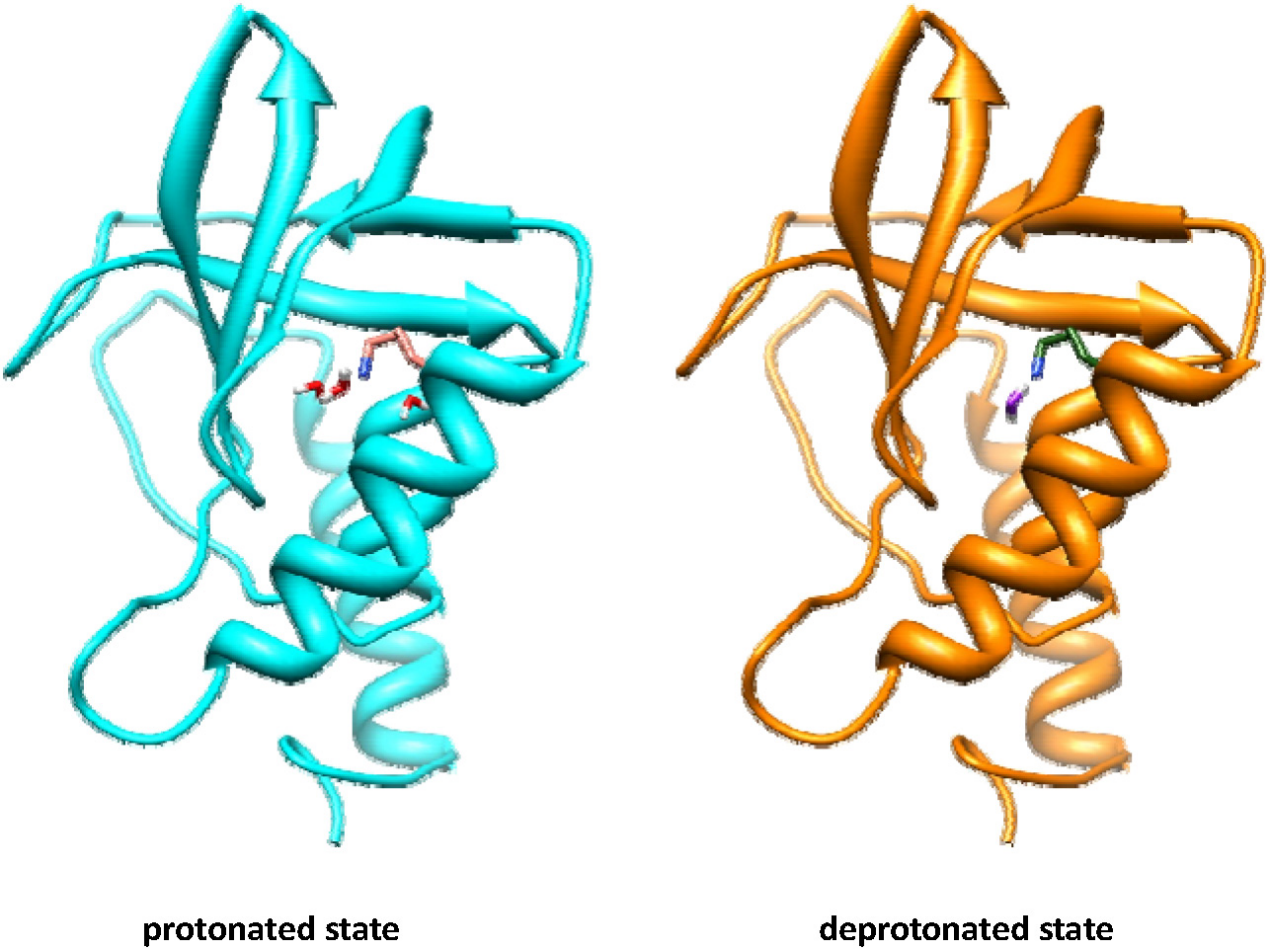
SNase Δ+PHS/V66K in the protonated and deprotonated states after 50 ns VMMS simulation. K66 sidechain has 3 hydrogen bonding water in the protonated state but only 1 hydrogen bonding water in the deprotonated state.

We examined the simulation trajectory to determine what causes the large deviation and fluctuation in K66’s pKa. First, we found that the protein conformation remained stable throughout the simulation. As shown in the rmsd plot of Fig 12, after the 100 ns simulation, the rmsds of the protein backbone and all atoms from the initial structure were about 1.4 Å and 2 Å, respectively. While certain conformational fluctuations occurred, the sidechain of K66 remained inside its original place, indicating that the large deviation of K66’s pKa is not necessarily due to its sidechain flipping in and out at different protonation states. Next, we examined the hydrogen bonding water molecules during the simulation. Because each state had its own solvent boxes, we can easily see the differences in solute-solvent interactions. In the middle panel of Fig 12, we plot the numbers of water molecules that hydrogen bond to the sidechain of K66. As can be seen, the VMMS simulation started with the same conformations for both states where there were 3 water molecules hydrogen bonding to K66’s sidechain. At 10 ns, the hydrogen bonded water in the deprotonated state reduced to 2 and the pKa reduced to between 7 and 8. At 20 ns the hydrogen bonded water of the deprotonated state reduced further to 1 and the pKa decreased to below 4. Then at 30ns, the hydrogen bonding water of the deprotonated state increased to 2 and the pKa reached over 7 again. At 40 ns, the hydrogen bonding water of the deprotonated state reduced to 1 and the pKa value reduced to slightly below the experiment value of 5.6. This tight correlation between the hydrogen bonding water number and the pKa value strongly suggests that water penetration around the K66 sidechain plays an important role in the large deviation of K66’s pKa. This result agrees with the high dielectric constant observed in the interior of this protein[40]. Fig 13 shows the protein and the hydrogen bonding water in both states.

The large fluctuation in the pKa of lysine66 shown in Fig 12 indicates that much longer simulation is needed to provide an accurate pKa value. This short simulation only provides a preliminary insight of the system. Thorough understanding of the system is beyond the scope of this paper.

This example shows the advantage to have explicit solvent for each protonation state in the VMMS method. For single state simulation methods, for example, λ-dynamics, conformations are sampled in a serial manner. The free energy difference between two states can only be calculated after a significant number of transitions between states. While in VMMS, both states are sampled simultaneously, and the free energy difference can be estimated right away. The most striking benefit is when the energy barrier for state transition is high, the VMMS method does not suffer the energy barrier crossing difficulty. In addition, the VMMS method can be combined with other existing accelerated simulation methods, such as the self-guided Langevin dynamics[41–43] to reach the conformation favoring dominant states quickly.

The protonation equilibrium is especially suitable for the VMMS method. By forcing the solute to sample the overlapping conformational space of different states, the VMMS method can accurately estimate the free energy differences between these states. However, when the solute conformation spaces of some states are not completely accessible by other states, alternate techniques or companion methods need to be employed to properly sample all important conformational spaces. Therefore, the VMMS method of current version is limited to cases where the solute conformation of one state is fully accessible by other states. In addition to protonation equilibrium, another such case is oxidation-reduction equilibrium where the oxidized and reduced states are different only by charges.

## Discussions

This work presents the VMMS simulation method for directly simulating the equilibrium of multiple chemical states. Through including explicit solvent environments of all states, this method avoids the time-consuming solvent reorientation between states, allowing free energy differences between different states to be efficiently estimated during simulation. Using model systems, we examined the conformational distribution of the VMMS ensemble and validated the reweighting formula from the VMMS distributions to pure state distributions.

The VMMS method is applied here to study protonation equilibrium. At a given pH, the VMMS simulation produces equilibrium molar fractions of all states. These molar fractions in turn will affect the potential energy of the VMMS solute and the conformational search. pKa can be determined by identifying the pH where the protonated and deprotonated states have equal molar fractions.

For systems with many titration sites, we employ implicit sites to reduce the number of subsystems in a VMMS simulation. This implicit site treatment allows VMMS simulations scale linearly with the number of titration sites. Through the heptapeptide we demonstrated that the VMMS–1 simulation with one explicit site and two implicit sites produced very similar result to the VMMS simulation with all explicit sites. We applied VMMS–1 simulations to evaluated pKas of 9 titration sites in mouse epidermal growth factor and the results agree qualitatively with the NMR measurement[36].

Applying the VMMS method to SNase Δ+PHS/V66K in explicit water, we found that the large deviation in lysine66’s pKa is likely due to the state-dependent penetration of water into the protein interior. This example demonstrates the VMMS method can properly handle explicit water for this difficult case in pKa calculation.

The VMMS method provides a general approach to study multistate equilibrium. Unlike existing simulation methods that either study state equilibrium indirectly or sample different states sequentially, the VMMS method simulates all states simultaneously and utilizes experiment observable molar fractions as state variables in simulation. It is expected that the VMMS method will find more applications in biological problems related to the equilibrium of multiple states.

## Methods

### The VMMS system

**Fig 14:**
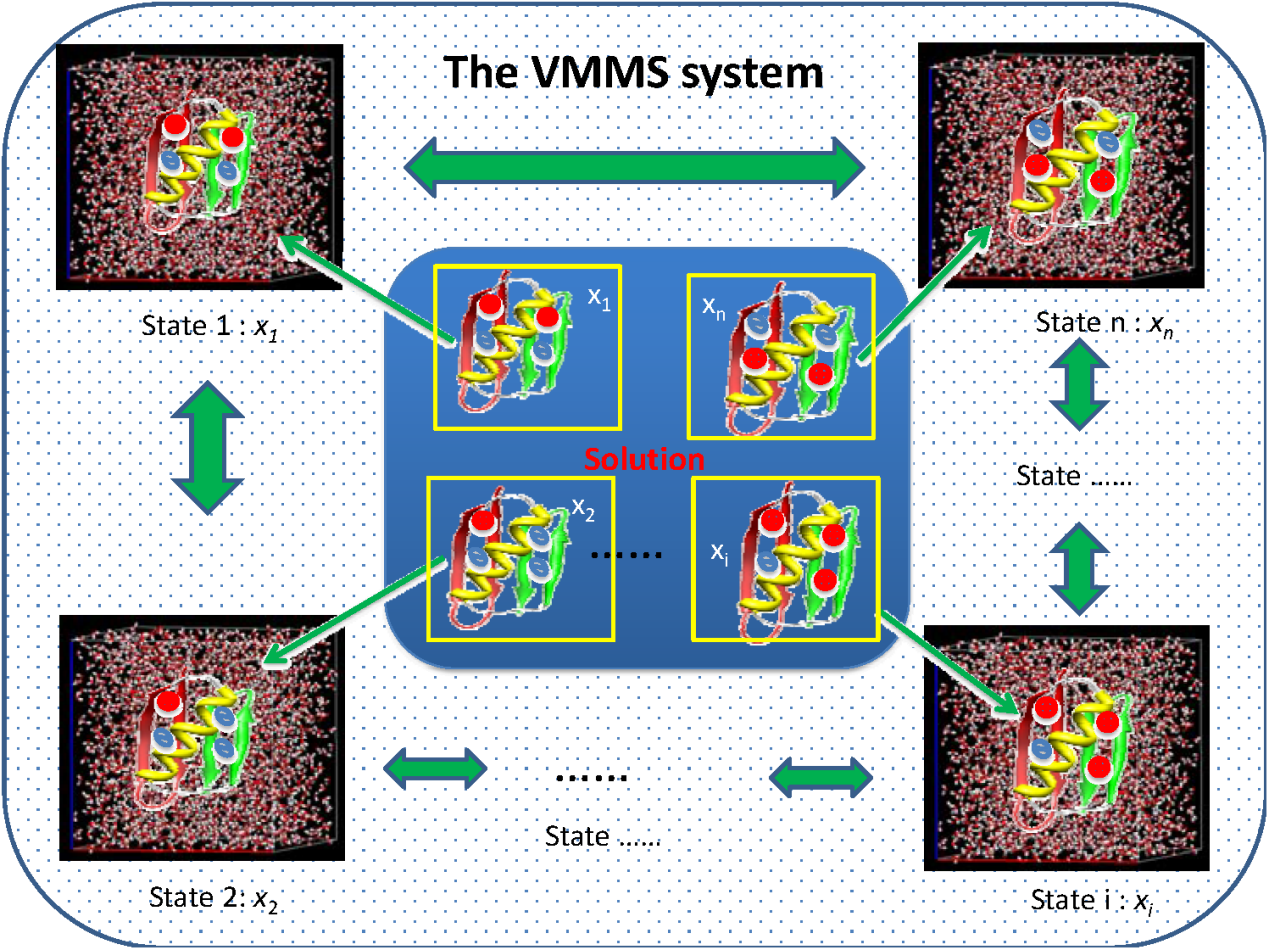
State equilibrium is represented as a virtual mixture of multiple states (VMMS). In a solution shown in the center, all states equilibrate with each other and exist by state molar fractions, *x*_1_, *x*_2_,⋯, *x*_*n*_. In VMMS, every state is explicitly represented by an all-atom subsystem. Each state has its own solvent environment and solvent can be either implicit or explicit. All subsystems form a virtual ideal solution to equilibrate with each other.

We design a VMMS system to simulate state equilibrium of a solute in a solvent environment, implicit or explicit. Consider a solute, e.g., a protein, in solution as shown in Fig 14. The solution (shown in the center) contains a solute in various chemical states, e.g., protonated and deprotonated states for ionizable residues. All chemical states of the solute coexist in the solution at equilibrated concentration. At the low concentration limit, the solute molecules in different chemical states do not interact with each other. Therefore, the solute at each state can be represented by a solvated molecule. In other words, every solute is solvated by its own solvent, and solvent molecules only interact with the solute of one state.

Because the conformational distribution and the chemical state of solute are interdependent, conformational sampling should be correlated to the equilibrium of chemical states. The state equilibrium depends on the state free energies, which can be calculated through adequate sampling of individual states, as well as the overlapping conformational space between states. In this work, we let solute of all states to share the same conformation to enhance the sampling on the overlapping region. On the other hand, we let solvents independent of each other to have adequate sampling of individual solvent states. Here, solvent plays a role to provide solvation to guide solute dynamic simulation. Even though solvent molecules explicitly surround the solute, they behave just like an implicit solvation model to provide solvation energy to the solute. In other words, we use the interaction from the explicit solvent to calculate solvation energy. Using explicit solvent to provide solvation effect for solute dynamic simulation has been explored previously[44]. This design avoids solvent reorientation upon state transition and allows the solute to undergo a state-equilibrated conformational search. The solvent is always in a state-equilibrated condition and provides more accurate solvation than an implicit solvation model.

If the solute has *n* chemical states, the VMMS system will have *n* subsystems, one for each state including the environment (solvent) around it. While the solute at each state has its own solvent environment, the solute molecules at all states share the same conformation and they search the conformational space together. In other words, each subsystem is a solute-solvent simulation box containing a solute surrounded by its implicit/explicit solvent environment. The solute molecules in all subsystems are constrained to have the same conformation and move the same way, but the solvent molecules are not constrained and move under their own interaction environment. The solvents sample their own conformational space to provide equilibrated solvation to their solute. This design makes it very convenient to calculate relative free energies of the states using Bennett’s ratio method [45]. Let’s image that the *n* states form an ideal solution and that each state has its own population defined as state molar fractions, *x*_1_, *x*_2_,⋯, *x*_*n*_, where *x*_1_ + *x*_2_ +⋯+ *x*_*n*_ = 1. At equilibrium, all states have the same chemical potential, which determine the equilibrium molar fractions of all states. Because these states are not physically mixed to form a real solution as in a mixture simulation[46], the system is the so-called virtual mixture of multiple states (VMMS).

In summary, a VMMS system contains one solute distributing in *n* states, *P*(*x*_1_, *x*_2_,⋯, *x*_*n*_), and *n* solvents, *W*_*i*_, *i* = 1,2,…,*n*, which provide solvation to the n states. The solute exists in all *n* chemical states by the state molar fractions of *x*_1_, *x*_2_,⋯, *x*_*n*_. Solvent *W*_*i*_ only interacts with the solute of state *i*. The VMMS system is denoted as: $P(x_{1},x_{2},\cdots ,x_{n})+\sum_{i}^{n}W_{i}$. We define a VMMS state *j*, denoted as *V*_*j*_, as the solute at the mixed states, *P*(*x*_1_, *x*_2_,⋯, *x*_*n*_), in solvent *W*_*j*_. The difference between a pure state *j* and a VMMS state *j* lays at the solute only. In a pure state, the solute exists only in one chemical state, i.e., *P*(*x*_*j*_ = 1, *x*_*i* ≠ *j*_ = 0), while in the VMMS state, *V*_*j*_, the solute exists in all chemical states, i.e., *P*(*x*_1_, *x*_2_,⋯, *x*_*n*_).

### VMMS conformational distribution

For a subsystem *j* containing a solute, P, of state *j* and solvent *W*_*j*_, the potential energy, *E* ^(*j*)^, can be separated into solute part, $E_{\text{P}}^{\left(j\right)}$, and solvent part, $E_{\text{W}}^{\left(j\right)}$:

$$E^{\left(j\right)}=E_{\text{P}}^{\left(j\right)}+E_{\text{W}}^{\left(j\right)}$$ (1)

The solute interaction is a sum of all pairwise interactions involving the solute, including solute-solute and solute-solvent interactions:

$$E_{\text{P}}^{\left(j\right)}=\sum_{a\in \text{P}}E_{\text{a}}^{\left(j\right)}=\frac{1}{2}\sum_{a\in \text{P}}\sum_{i}^{N^{\left(j\right)}}\varepsilon_{\text{ai}}^{\left(j\right)}$$ (2)

Here, *N* ^*(j)*^ is the number of atoms, $E_{\text{a}}^{\left(j\right)}$ is the total interaction energy of atom *a*, and $\varepsilon_{\text{ai}}^{\left(j\right)}$ is the interaction between atom *a* and atom *i* in subsystem *j*. Similarly, the solvent interaction is a sum of all pairwise interactions involving the solvent, including solvent-solvent and solute-solvent interactions:

$$E_{\text{W}}^{\left(j\right)}=\sum_{a\in {\text{W}}_{\text{j}}}E_{\text{a}}^{\left(j\right)}=\frac{1}{2}\sum_{a\in {\text{W}}_{\text{j}}}\sum_{i}^{N^{\left(j\right)}}E_{\text{ai}}^{\left(j\right)}$$ (3)

For a VMMS system, $P(x_{1},x_{2},\cdots ,x_{n})+\sum_{i}^{n}W_{i}$, there are *n* subsystems, one for each state. The VMMS system has the solvents of all subsystems, but only 1 solute, distributing in all states by the state molar fractions. Therefore, the VMMS potential energy has the following form:

$$E^{\left(\text{VMMS}\right)}=\sum_{i}^{n}\left(x_{i}E_{\text{P}}^{\left(i\right)}+E_{\text{W}}^{\left(i\right)}\right)$$ (4)

The VMMS state *j*, *V*_*j*_, contains the VMMS solute and the solvent interacting with the solute at state *j*. The potential energy of *V*_*j*_ is a sum of the solute potential energy and the solvent potential energy in subsystem *j*:

$$E^{\left({\text{V}}_{j}\right)}=\sum_{i}^{n}\left(x_{i}E_{\text{P}}^{\left(i\right)}\right)+E_{\text{W}}^{\left(j\right)}$$ (5)

which is different from the potential energy of the pure state *j*, Eq (1), in the solute part.

The partition function of the VMMS ensemble is:

$$Q^{\left(\text{VMMS}\right)}=\sum_{\Omega^{\left(\text{VMMS}\right)}}\text{exp}(-\beta (E^{\left(\text{VMMS}\right)})=\sum_{\Omega^{\left(\text{VMMS}\right)}}\prod_{i}^{n}\text{exp}(-\beta (x_{i}E_{\text{P}}^{\left(i\right)}+E_{\text{W}}^{\left(i\right)})$$ (6)

where Ω^(VMMS)^ represents the conformational space of the VMMS system and $\beta =\frac{1}{kT}$.

When *x*_*j*_ = 1 and *x*_*i* ≠ *j*_ = 0, the VMMS system becomes a pure state *j* plus all other solvents, *W*_*i* ≠ *j*_. The partition function can be written to the following form:

$$Q^{\left(\text{VMMS}\right)}(x_{j}=1,x_{i\neq j}=0)=\sum_{\Omega^{\left(\text{VMMS}\right)}}\text{exp}(-\beta (E_{\text{P}}^{\left(j\right)}+E_{\text{W}}^{\left(j\right)}))\prod_{i\neq j}^{n}\text{exp}(-\beta E_{\text{W}}^{\left(i\right)})$$ (7)

Therefore, we have the relation between VMMS ensembles at (*x*_1_, *x*_2_,⋯, *x*_*n*_) and at (*x*_*j*_ = 1, *x*_*i* ≠ *j*_ = 0):

$$\begin{array}{c} \frac{Q^{\left(\text{VMMS}\right)}(x_{j}=1)}{Q^{\left(\text{VMMS}\right)}(x_{1},x_{2},\cdots ,x_{n})}=\frac{\sum_{\Omega^{\left(\text{VMMS}\right)}}\text{exp}(-\beta \left(E_{\text{P}}^{\left(Vj\right)}+\sum_{i}^{n}E_{\text{W}}^{\left(i\right)}\right))}{\sum_{\Omega^{\left(\text{VMMS}\right)}}\text{exp}(-\beta \sum_{i}^{n}\left(x_{i}E_{\text{P}}^{\left(i\right)}+E_{\text{W}}^{\left(i\right)}\right))}\\ \;=\frac{\sum_{\Omega^{\left(\text{VMMS}\right)}}\text{exp}(-\beta (E_{\text{P}}^{\left(j\right)}-\sum_{i}^{n}x_{i}E_{\text{P}}^{\left(i\right)}))\text{exp}(-\beta \sum_{i}^{n}\left(x_{i}E_{\text{P}}^{\left(i\right)}+E_{\text{W}}^{\left(i\right)}\right))}{\sum_{\Omega^{\left(\text{VMMS}\right)}}\text{exp}(-\beta \sum_{i}^{n}\left(x_{i}E_{\text{P}}^{\left(i\right)}+E_{\text{W}}^{\left(i\right)}\right))}\\ \;=<\text{exp}(-\beta \left(E_{\text{P}}^{\left(j\right)}-\sum_{i}^{n}x_{i}E_{\text{P}}^{\left(i\right)}\right){>}_{\text{VMMS}}\\ \;=<\text{exp}(-\beta \left(E_{}^{\left(j\right)}-E_{}^{\left(Vj\right)}\right){>}_{\text{VMMS}} \end{array}$$ (8)

Eq (8) shows that the VMMS ensemble average of property *P* at (*x*_*j*_ = 1, *x*_*i* ≠ *j*_ = 0) can be obtained in a VMMS simulation at (*x*_1_, *x*_2_,⋯, *x*_*n*_) through reweighting by a factor:

$$w_{j}=\exp(-\beta (E^{\left(\text{j}\right)}-E^{\left(Vj\right)}))=\text{exp}(-\beta \sum_{i}^{n}x_{i}(E_{\text{P}}^{\left(\text{j}\right)}-E_{\text{P}}^{\left(i\right)}))$$ (9)

Because at (*x*_*j*_ = 1, *x*_*i* ≠ *j*_ = 0) the solute energy depends only on solvent *W*_*j*_ and is independent of all other solvents, it is easy to prove that the VMMS ensemble average of any state *j* related property, *P*_*j*_, at (*x*_*j*_ = 1, *x*_*i* ≠ *j*_ = 0) equals the canonical ensemble average at pure state *j*:

$$\begin{array}{c} <P_{j}{>}_{\text{VMMS}(x_{j}=1)}=\frac{\sum_{\Omega^{\left(\text{VMMS}\right)}}P_{j}\text{exp}(-\beta (E_{\text{P}}^{\left(j\right)}+E_{\text{W}}^{\left(j\right)}))\prod_{i\neq j}^{n}\text{exp}(-\beta E_{\text{W}}^{\left(i\right)})}{\sum_{\Omega^{\left(\text{VMMS}\right)}}\text{exp}(-\beta (E_{\text{P}}^{\left(j\right)}+E_{\text{W}}^{\left(j\right)}))\prod_{i\neq j}^{n}\text{exp}(-\beta E_{\text{W}}^{\left(i\right)})}\\ \;=\frac{\sum_{\Omega^{\left(\text{VMMS}\right)}}P_{j}\text{exp}(-\beta (E_{\text{P}}^{\left(j\right)}+E_{\text{W}}^{\left(j\right)}))\prod_{i\neq j}^{n}\text{exp}(-\beta E_{\text{W}}^{\left(i\right)})}{\sum_{\Omega^{\left(\text{VMMS}\right)}}\text{exp}(-\beta (E_{\text{P}}^{\left(j\right)}+E_{\text{W}}^{\left(j\right)}))}\frac{\sum_{\Omega^{\left(\text{VMMS}\right)}}\text{exp}(-\beta (E_{\text{P}}^{\left(j\right)}+E_{\text{W}}^{\left(j\right)}))}{\sum_{\Omega^{\left(\text{VMMS}\right)}}\text{exp}(-\beta (E_{\text{P}}^{\left(j\right)}+E_{\text{W}}^{\left(j\right)}))\prod_{i\neq j}^{n}\text{exp}(-\beta E_{\text{W}}^{\left(i\right)})}\\ \;=\frac{<P_{j}\prod_{i\neq j}^{n}\text{exp}(-\beta E_{\text{W}}^{\left(i\right)}){>}_{\text{j}}}{<\prod_{i\neq j}^{n}\text{exp}(-\beta E_{\text{W}}^{\left(i\right)}){>}_{\text{j}}}=\frac{<P_{j}{>}_{\text{j}}<\prod_{i\neq j}^{n}\text{exp}(-\beta E_{\text{W}}^{\left(i\right)}){>}_{\text{j}}}{<\prod_{i\neq j}^{n}\text{exp}(-\beta E_{\text{W}}^{\left(i\right)}){>}_{\text{j}}}=<P_{j}{>}_{\text{j}} \end{array}$$

The separation of *P*_*j*_ and $\prod_{i\neq j}^{n}\text{exp}(-\beta E_{\text{W}}^{\left(i\right)})$ is because they are independent of each other at (*x*_*j*_ = 1, *x*_*i* ≠ *j*_ = 0). Therefore, the ensemble average of any state *j* related property, *P*_*j*_, in pure state *j* can be reweighted from a VMMS simulation by:

$$<P_{j}{>}_{j}=<P_{j}{>}_{\text{VMMS}(x_{j}=1)}=\frac{<P_{j}w_{j}{>}_{\text{VMMS}}}{<w_{j}{>}_{\text{VMMS}}}$$ (10)

### Using 3D IPS in VMMS simulation

The reweighting in VMMS simulation as shown in Eqs (9) and (10) need solute energies, which can be efficiently estimated from pairwise interactions by Eq (2). The isotropic periodic sum (IPS) method provides a pairwise way to accurately calculate long range interactions[24,47–50]. The use of IPS here can simplify or eliminate some of the problems encountered with Ewald methods. Specifically, the use of IPS allows the partition of energy components in a manner that is not possible with PME, unless a separate PME calculation is done for each state and for solute and solvent separately. In the result section, we validate the IPS accuracy through free energy calculation of model compounds.

The IPS potential for polar electrostatic interaction is[24]:

εeleIPSp(r,Rc)={qiqjr(1−1916(rRc)+3516(rRc)3−2116(rRc)5+516(rRc)7)r≤Rc0r>Rc (11)

where *R*_*c*_ is the local region radius, commonly called cutoff distance. *q*_*i*_ and *q*_*j*_ are charges at atoms *i* and *j*, respectively. The total electrostatic interaction energy can be calculated as simple as typical cutoff methods:

$$E_{\text{ele}}^{\left(\text{IPS}\right)}(r^{N},R_{c})=\frac{1}{2}\sum_{i}^{N}\sum_{j}^{N}\varepsilon_{\text{ele}}^{\left(\text{IPSp}\right)}(r_{ij},R_{c})=\frac{1}{2}\sum_{i}^{N}\sum_{r_{ij}<R_{c}}\varepsilon_{\text{ele}}^{\left(\text{IPSp}\right)}(r_{ij},R_{c})$$ (12)

Here, we use the neutral charge assumption so that the total IPS boundary electrostatic energy is zero. For Lennard-Jones (L-J) energy, the IPS potentials for the dispersion and repulsive parts are:

$$\varepsilon_{\text{disp}}^{3\text{D}-\text{IPS}}(r,R_{\text{c}})\approx -\frac{C}{r^{6}}(\;1+\frac{7}{16}{\left(\frac{r}{R_{\text{c}}}\right)}^{6}+\frac{9}{14}{\left(\frac{r}{R_{\text{c}}}\right)}^{8}-\frac{3}{28}{\left(\frac{r}{R_{\text{c}}}\right)}^{10}+\frac{6}{7}{\left(\frac{r}{R_{\text{c}}}\right)}^{12})$$ (13)

$$\varepsilon_{\text{rep}}^{3\text{D}-\text{IPS}}(r,R_{\text{c}})\approx \frac{A}{r^{12}}(\;1+\;\frac{5}{787}{\left(\frac{r}{R_{\text{c}}}\right)}^{12}+\;\frac{9}{26}{\left(\frac{r}{R_{\text{c}}}\right)}^{16}-\;\frac{3}{13}{\left(\frac{r}{R_{\text{c}}}\right)}^{20}+\frac{27}{26}{\left(\frac{r}{R_{\text{c}}}\right)}^{24})$$ (14)

Here *C* and *A* are dispersion and repulsion constants, respectively, for L-J potential. The pairwise L-J IPS potential is:

εLJ3D-IPSp(r,Rc)={εdisp3D-IPSp(r,Rc)+εrep3D-IPSp(r,Rc)−εdisp3D-IPSp(Rc,Rc)−εrep3D-IPSp(Rc,Rc)r≤Rc0r>Rc (15)

And the total L-J interaction is a sum over all atom pairs:

$$E_{\text{LJ}}^{\left(\text{IPS}\right)}(r^{N},R_{c})=\frac{1}{2}\sum_{i}^{N}\sum_{r_{ij}<R_{c}}\varepsilon_{\text{LJ}}^{\left(\text{IPS}\right)}(r_{ij},R_{c})+\frac{4\pi R_{c}^{3}}{3NV}\sum_{i}^{N}\sum_{j}^{N}\left(\varepsilon_{\text{disp}}^{\left(\text{IPS}\right)}(R_{c},R_{c})+\varepsilon_{\text{rep}}^{\left(\text{IPS}\right)}(R_{c},R_{c})\right)$$ (16)

The first term is the pairwise IPS potential and the second term is the L-J IPS boundary energy, which is constant during a *NVT* simulation.

Using pairwise potentials is not only convenient for separating solute and solvent interactions, required by Eqs (1–3), but also helps avoid repeated calculation for the same conformation at different states, which is essential for free energy calculation. For the same conformation, energies of different states can be calculated efficiently with a small amount of overhead:

$$E_{\text{ele}}^{\left(\text{IPSp}\right)}(q^{\left(k\right)})=\frac{1}{2}\sum_{i}^{N_{\text{nc}}}\sum_{j}^{N_{\text{nc}}}\varepsilon_{\text{ele}}^{\left(\text{IPSp}\right)}(r_{ij},R_{c})+\sum_{i}^{N_{c}}q_{i}^{\left(k\right)}\sum_{j}^{N_{\text{nc}}}q_{j}{\hat{\varepsilon }}_{\text{ele}}^{\left(\text{IPSp}\right)}(r_{ij},R_{c})+\frac{1}{2}\sum_{i}^{N_{c}}q_{i}^{\left(k\right)}\sum_{j}^{N_{\text{c}}}q_{j}^{\left(k\right)}{\hat{\varepsilon }}_{\text{ele}}^{\left(\text{IPSp}\right)}(r_{ij},R_{c})$$ (17)

where *N*_nc_ is the number of non-changing atoms and *N*_c_ is the number of changing atoms, ${\hat{\varepsilon }}_{ij}$ is the unit charge interaction, ${\hat{\varepsilon }}_{ij}=\varepsilon_{ij}/(q_{i}q_{j})$, and $q_{i}^{\left(k\right)}$ is the charge of atom *i* at state *k*. The first term is for atom pairs of unchanged charges, which costs the most, and does not need to be recalculated for different charge states.

### Free energy differences between states

The VMMS simulation samples conformations at all states, providing sufficient information to calculate free energy differences between all states. Because the solute is not in either of the pure states, reweighting is needed to obtain conformational distributions at the pure states.

**Fig 15:**
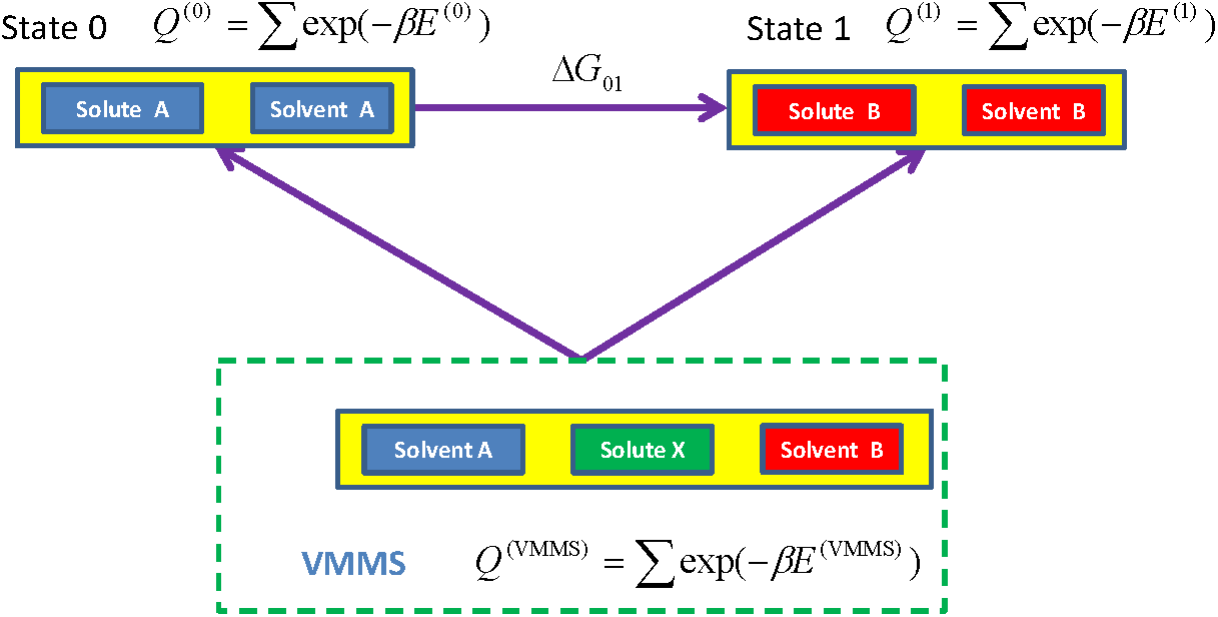
Calculation diagram for state free energy differences. The conformational distributions of state 0 and 1 can be obtained through reweighting from the VMMS simulation.

Fig 15 shows the free energy calculation diagram. To calculate free energy between state 0 and state 1, we reweight the VMMS conformation distribution to state 0 and state 1 so that we can apply Bennett’s ratio method[45]. Here, we modify Bennett’s Ratio method for the VMMS simulation:

$$\Delta G_{01}=C_{01}-kT\;\text{ln}\frac{<f(\beta (E^{\left(1\right)}-E^{\left(0\right)}-C_{01}))\text{exp}(-\beta (E^{\left(0\right)}-E^{\left(V0\right)}){>}_{\text{VMMS}}}{<f(\beta (E^{\left(0\right)}-E^{\left(1\right)}+C_{01}))\text{exp}(-\beta (E^{\left(1\right)}-E^{\left(V1\right)}){>}_{\text{VMMS}}}\frac{<\text{exp}(-\beta (E^{\left(1\right)}-E^{\left(V1\right)}){>}_{\text{VMMS}}}{<\text{exp}(-\beta (E^{\left(0\right)}-E^{\left(V0\right)}){>}_{\text{VMMS}}}$$ (18)

where the Fermi function is defined as:

$$f(x)=\frac{1}{1+\text{exp}(x)}$$ (19)

The VMMS simulation can be performed with enhanced sampling methods, such as self-guided Langevin dynamics[41,42,51,52], to accelerate the conformation search. When applying SGLD, the SGLD reweighting factors, *w*_SG0_ and *w*_SG1_, should be incorporated into Eq (18):

$$\Delta G_{01}=C_{01}-kT\;\text{ln}\frac{<f(\beta (E^{\left(1\right)}-E^{\left(0\right)}-C_{01}))w_{\text{SG}0}\text{exp}(-\beta (E^{\left(0\right)}-E^{\left(V0\right)}){>}_{\text{VMMS}}}{<f(\beta (E^{\left(0\right)}-E^{\left(1\right)}+C_{01}))w_{\text{SG}1}\text{exp}(-\beta (E^{\left(1\right)}-E^{\left(V1\right)}){>}_{\text{VMMS}}}\frac{<w_{\text{SG}1}\text{exp}(-\beta (E^{\left(1\right)}-E^{\left(V1\right)}){>}_{\text{VMMS}}}{<w_{\text{SG}0}\text{exp}(-\beta (E^{\left(0\right)}-E^{\left(V0\right)}){>}_{\text{VMMS}}}$$ (20)

This equation provides a useful connection between an accelerated VMMS simulation and the desired canonical ensemble. If choosing SGLDfp[42] or SGLD-GLE[43] where *w*_SG_ = 1, one can simply use Eq (18) to calculate relative free energies.

### State equilibrium

In VMMS, all states form a virtual solution at equilibrium molar fractions. The chemical potential of each state depends on its molar fraction:

$$\mu_{i}=\frac{\partial G}{\partial n_{i}}=\mu_{i}^{0}+kT\;\ln\;x_{i}$$ (21)

Where $\mu_{i}^{0}$ is the standard chemical potential of state i. At equilibrium, the chemical potential of all states equal: *μ*_*i*_ = *μ*_*j*_, therefore, we have:

$$\frac{x_{j}}{x_{i}}=\text{exp}(-\frac{\mu_{j}^{0}-\mu_{i}^{0}}{kT})=\text{exp}(-\frac{\Delta \mu_{ij}^{0}}{kT})$$ (22)

For multiple states, we use the following formula to derive molar fractions from the standard chemical potential differences:

xk=(∑jnexp(−Δμkj0kT))−1∑in(∑jnexp(−Δμij0kT))−1 (23)

For protonation equilibrium, the standard chemical potential difference contains many contributions such as those from molecular interactions and those from quantum mechanics of protonation. All contributions other than those from molecular interactions are assumed to be constant for a given titration site and can be estimated from the equilibrium properties of model compounds. More details can be found elsewhere.[1,3–6,53] At a given pH, the standard chemical potential difference of deprotonation can be calculated from the state free energy difference:

$$\Delta \mu_{ij}^{0}=\Delta G_{ij}-kT(pH-pK{\text{a}}^{\text{ref}})\text{ln}\;10-\Delta G_{ij}^{\text{ref}}$$ (24)

Here, ${\text{pKa}}_{ij}^{\text{ref}}$ is the experimental pKa of a model compound for the corresponding amino acid to change from the protonated state, *i*, to the deprotonated state, *j*, and $\Delta G_{ij}^{\text{ref}}$ is the free energy difference calculated from simulation of the model compound. Using Eqs (23) and (24), one can calculate equilibrium molar fractions of all states. Based on the definition of pKa, from equilibrium molar fractions we can calculate pKa:

$$\text{p}K\text{a}=-\text{lg}\frac{x_{\text{D}}[H]}{x_{\text{H}}}=\text{pH}+\text{p}x_{\text{D}}-\text{p}x_{\text{H}}$$ (25)

where *x*_H_ and *x*_D_ are the molar fractions of the protonated and deprotonated states, respectively. Typically, in constant pH simulation, pKa is determined by the pH value where *x*_H_ = *x*_D_. pKa can also be directly calculated from a VMMS simulation at equal state molar fractions (*x*_H_ = *x*_D_) where $\Delta \mu_{ij}=\Delta \mu_{ij}^{0}=0$ and p*K*a = pH. From Eq (24), we have:

$$\text{p}K\text{a}=\text{p}K{\text{a}}^{\text{ref}}+\frac{\Delta G(x_{\text{H}}=x_{\text{D}})-\Delta G^{\text{ref}}}{kT\;\text{ln}\;10}$$ (26)

### Using implicit sites to handle systems with a large number of titration sites

VMMS uses explicit chemical states to efficiently sample the conformations at these states and avoid difficulties related to state transitions. Because each titration site has two states, for a system with *m* titration sites there would be as many as 2^*m*^ states. Obviously, it is impractical to include explicitly all protonation states in a VMMS simulation when *m* is large, say, more than 6. To circumvent this burden, we propose a divide-and-conquer approach to explicitly consider only one or a few titration sites at a time and treat the remaining sites implicitly to have combined states. This implicit site approximation is the same, in spirit, to the titration coordinate when running λ-dynamics for constant pH simulations[4,6], where the titration coordinate λ represents a combination of protonated and deprotonated states. In other words, in λ-dynamics all ionizable sites are treated as a mixture of 1-λ protonated and λ deprotonated states, just like an implicit site described here. The titration of an ionizable site in λ-dynamics corresponds to a transition of λ between 0 and 1. But in VMMS, the titration of an ionizable site corresponds to a change of molar fractions, which only happens when an ionizable site is explicit.

A titration site is explicit when it has distinct protonated and deprotonated states, which differ by their atomic charges. To make a titration site implicit, its protonated and deprotonated states are converted to a single state where the atomic charges are a linear combination of the charges in the two explicit states.

$$q_{a}^{\left(\text{im}\right)}=(1-x_{\text{D}})q_{a}^{\left(\text{H}\right)}+x_{\text{D}}q_{a}^{\left(\text{D}\right)}.$$ (27)

Here, $q_{a}^{\left(\text{H}\right)},\;q_{a}^{\left(\text{D}\right)}$ are atomic charges of atom *a* in the protonated and deprotonated states, respectively, and $q_{a}^{\left(\text{im}\right)}$ is the atomic charge in the implicit site. When a site is implicit, all atoms in the site are implicit and their charges are defined by Eq (27). When a titration site changes from implicit to explicit, its atomic charges return to $q_{a}^{\left(\text{H}\right)}$ for the protonated state and to $q_{a}^{\left(\text{D}\right)}$ for the deprotonated state.

During a VMMS simulation, one or a few titration sites are chosen to be explicit and the rest implicit. The molar fractions of the explicit sites are updated during simulation according to Eqs (23) and (24). After this explicit simulation period, the explicit sites are converted to implicit based on Eq (27) and one or a few other implicit sites are chosen to be converted to explicit for the next explicit simulation period. This process can loop repeatedly through all titration sites until the molar fractions of all sites converge.

For a system of *m* titration sites, a VMMS simulation can be carried out with *k* explicit titration sites (0<*k≤m*) and *m-k* implicit titration sites. We use VMMS-*k* to characterize a VMMS simulation by the number of explicit sites. Without mentioning the explicit site number, a VMMS simulation means a VMMS-*m* simulation where all titration sites are explicit, *k = m*. For example, for a protein of 3 titration sites, a VMMS–1 simulation means that at any moment during the simulation, there is one explicit site and two implicit sites, while a VMMS–3 or VMMS simulation means that all 3 sites are explicit. For a VMMS-*k* simulation, the system contains 2^*k*^ subsystems, one for each explicit state. Because the *k* explicit sites will loop through the *m* sites, the cost of a VMMS-*k* simulation is *O*(2^*k*^ *m* / *k*), which scales linearly with the number of titration sites, *m*. This implicit site treatment is optimal for parallel computing where different explicit sites can be simulated in parallel and their results, *x*_D_, will be used to update the atomic charges according to Eq (27) for simulations where the sites are implicit.

### Simulation details

The VMMS method is carried out as parallel simulations of a series of subsystems, one for each explicit chemical state. Energies and forces are calculated as in normal dynamic simulations. Before integrating the equation of motion, the solute forces are replaced by the weighted combination according to the state molar fractions, while the solvent forces remain unmodified:

$$f_{a}^{\left(\text{VMMS}\right)}=\{\begin{array}{cc} \sum_{i}^{n}\left(x_{i}f_{a}^{\left(i\right)}\right) & a\in \text{P}\\ f_{a}^{\left(i\right)} & a\notin \text{P} \end{array}$$ (28)

Using the VMMS forces to integrate the equation of motion for either MD, LD, or SGLD simulations, we propagate the conformation to sample the conformational space. The VMMS forces shown in Eq (28) will not conserve the total energy for each subsystem. To maintain energy conservation for each subsystem, we scale the solvent velocities in the following way:

$$v_{a}^{\left(i\right)}=\{\begin{array}{cc} {v^{\prime }}_{a}^{\left(i\right)} & \;a\in P\\ \; & \;\\ {v^{\prime }}_{a}^{\left(i\right)}\sqrt{1-\frac{\delta^{\left(i\right)}}{\sum_{a}^{Wi}\frac{1}{2}m_{a}v_{a}^{2}}} & \;a\notin P \end{array}$$ (29)

where ${v^{\prime }}_{a}^{\left(i\right)}$ is the velocity after current time step and $\delta^{\left(i\right)}=\sum_{a\in \text{P}}(f_{a}^{\left(V\text{MMS}\right)}-f_{a}^{\left(i\right)}){v^{\prime }}_{a}^{\left(i\right)}\delta t$ is the extra work of the VMMS forces do to subsystem *i*. The solute experiences the same forces and moves exactly the same way in all subsystems. To avoid numerical errors, solute movement is performed only in one subsystem and the coordinates are broadcasted to all subsystems. Other than these, all procedures are the same as that in regular dynamics simulations.

All simulations presented here were performed with CHARMM[54,55]. The all-atom CHARMM force field[56] was used for energy calculation. Excepted noted otherwise, all simulations were performed in a constant volume and a constant temperature of 300K using the SGLD-GLE method[43] with a local averaging time of *t*_L_ = 0.2 ps, a guiding factor of λ = 1, and a friction constant of ξ = 10/ps. A time step of 1 fs was used and SHAKE algorithm[57] was employed to fix the hydrogen connecting bond lengths.

Six typical ionizable residues as well as two terminal groups were examined here. They are aspartic acid (ASP or D), glutamic acid (GLU or E), lysine (LYS or K), arginine (ARG or R), histidine (HIS^δ^ and HIS^ε^ or H^δ^ and H^ε^), and tyrosine (TYR or Y), N-terminal group (Nter) and C-terminal group (Cter). Their model compounds were built by adding an acetyl (ACE) group to the N-terminal and a methyl amide (NME) group to the C-terminal, except for the terminal groups whose model compounds were built by attaching them to an alanine residue at one terminal and a block group at the other terminal.

**Fig 16:**
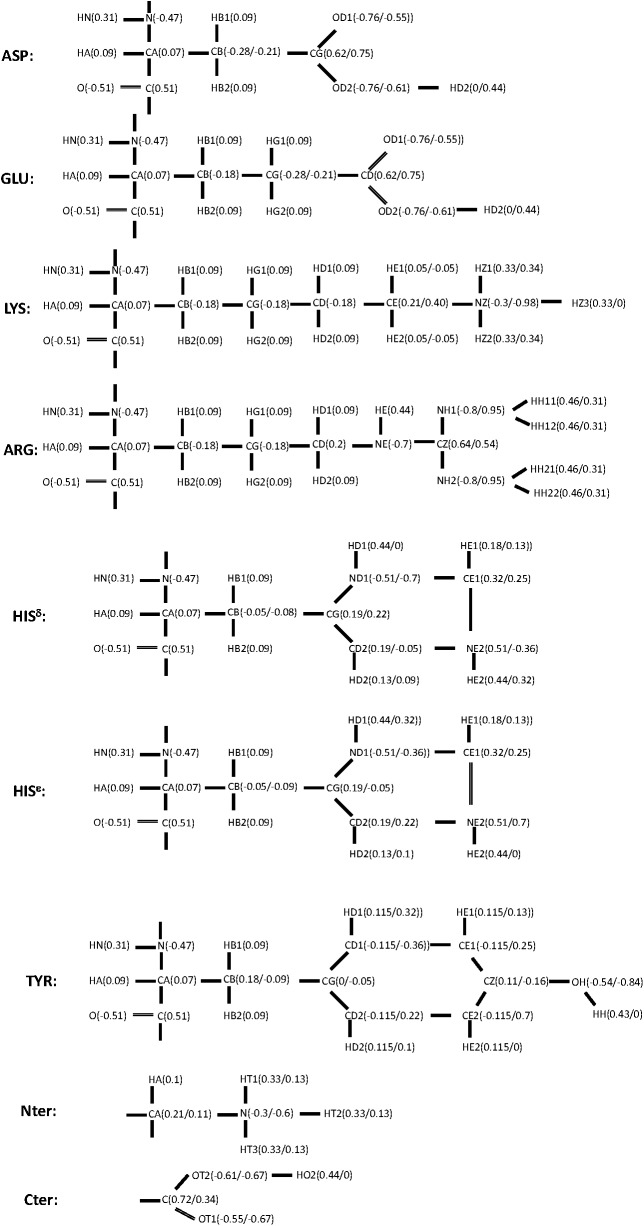
Atomic charges in the ionizable residues in the protonated/deprotonated states. Dummy hydrogen atoms with zero charge are added in the deprotonated states.

The deprotonated states of ionizable residues have a dummy hydrogen so that both states have the same number of atoms. The dummy hydrogen atom is identical to a hydrogen atom except that it has no charge. Fig 16 shows the atomic charges of these ionizable residues in their protonated and deprotonated states.

The VMMS systems were built by immersing a solute molecule in a box of water and any water whose oxygen distance to a solute atom was less than 2.4 angstroms was removed. It should be noted that different states can have different number of solvent molecules and/or ions. In this work, the same starting conformation was used for all states. During VMMS simulations, the Fermi functions and related weighting factors in Eq (18) were evaluated every 10 fs and free energies were calculated every 0.5 ps. During simulations, we used the following scheme to obtain local average free energies:

$${\tilde{P}}_{n}=(1-\frac{1}{L}){\tilde{P}}_{n-1}+\frac{1}{L}P_{n}$$ (30)

where L is the local averaging period. In the simulations reported here, we used *L* = 200.

For VMMS simulations at constant pH, molar fractions were updated every 0.5 ps according to Eq (23). For VMMS–1 simulations, one ionizable site was explicitly simulated for 10 ps before being converted to implicit. Of the 10 ps explicit simulation period, 2 ps were used for equilibrium and the remaining 8 ps were used for free energy and state molar fraction calculation. When a site was implicit, its state molar fractions remained unchanged.
